# Titanium and Vanadium Complexes of Tridentate Phenoxy-Imine and Phenoxy-Amine Ligands and Their Application in the Ring-Opening Polymerization of Cyclic Esters

**DOI:** 10.3390/molecules29010087

**Published:** 2023-12-22

**Authors:** Marzena Białek, Alicja Klimasińska, Grzegorz Spaleniak, Błażej Dziuk

**Affiliations:** 1 Institute of Chemistry, University of Opole, Oleska 48, 45-052 Opole, Polandgrzegorz.spaleniak@uni.opole.pl (G.S.); 2 Faculty of Chemistry, Wrocław University of Science and Technology, Wybrzeże Wyspiańskiego 27, 50370 Wrocław, Poland; blazej.dziuk@pwr.edu.pl

**Keywords:** ring-opening polymerization, *rac*-lactide, *L*-lactide, *ε*-caprolactone, titanium complex, vanadium complex, tridentate phenoxy-imine ligand, tridentate phenoxy-amine ligand

## Abstract

Phenoxy-imine and phenoxy-amine proligands, with the additional OH donor groups 2,4-*^t^*Bu_2_-6-(2-CH_2_(OH)-C_6_H_4_N=CH)C_6_H_3_OH (**L^1^H_2_**), 6-(2-CH_2_(OH)-C_6_H_4_N=CH)C_6_H_3_OH (**L^2^H_2_**), and 2,4-*^t^*Bu_2_-6-(2-CH_2_(OH)-C_6_H_4_NH-CH)C_6_H_3_OH (**L^3^H_2_**), were synthesized and their titanium (**Ti-L^1^**–**Ti-L^3^**) and vanadium (**V-L^1^**–**V-L^2^**) complexes were prepared in reactions with Ti(O*^i^*Pr)_4_ and VO(O*^i^*Pr)_3_, respectively. All new compounds were characterized with the use of FTIR, ^1^H, and ^13^C NMR spectroscopy; X-ray crystallography was also used to study proligands. All the complexes proved to be active catalysts in the ring-opening polymerization (ROP) of *ε*-caprolactone, *rac*-lactide, and *L*-lactide in the melt. The effects of the complex structure (transition metal type, presence of *t*Bu substituents, and type of nitrogen donor group), as well as the polymerization time and temperature, on the monomer conversion and polymer properties were investigated in detail.

## 1. Introduction

Polylactide (PLA) and poly(*ε*-caprolactone) (PCL) have received a lot of attention over the last few years due to their biodegradability and attractive physical and mechanical properties [1,2,3]. The microstructures, physical-chemical properties, possible applications, and synthesis methods of these polymeric materials have been discussed in detail in a number of review papers, for example, [4,5,6,7,8,9,10,11,12,13,14,15,16,17,18,19,20,21,22,23,24]. The common method for the synthesis of PLA and PCL is the ring-opening polymerization (ROP) of corresponding monomers, e.g., *ε*-caprolactone (*ε*-CL) and lactide (LA) monomers, with the use of structurally well-defined metal complexes that contain an initiating nucleophile, most commonly the alkoxy group OR, and a variety of ancillary ligands [2,5,25,26]. This method is regarded as the most efficient method for the synthesis of polyesters with well-controlled molar mass, composition, and microstructure [21]. The literature survey showed that titanium complexes, which contain mixed nitrogen/oxygen donor ligands, make one of the groups that very effectively promote the polymerization of cyclic esters [5,26]. They can be easily obtained in a one-step reaction of proligands with commercially available titanium tetra-alkoxide precursors. The low toxicity of titanium is another advantage that supports the use of titanium complexes in the polymerization of cyclic esters, especially in the synthesis of polymers, which are potentially intended for biomedical outlets [27,28]. Since the catalytic performance of metal complexes is largely determined by their ancillary ligands, the design of those ligands is also of great importance in the polymerization of lactones and lactides. Several titanium catalysts supported by new bi-, tri-, and tetradentate ligands with O donor atoms derived from hydroxyl group(s), and with N donor atoms coming from imine, amine, or other moieties, have been developed over the past five years or so. Ou et al. applied complexes based on [ONO]-tridentate Schiff bases in the ROP of cyclic esters and they showed that the electron-donating substituents on the ligands could drastically increase the catalytic activity in LA polymerization; that effect, however, was not observed for *ε*-CL polymerization [29]. As regards the ROP of cyclic esters in the presence of [ONN]-chelated titanium alkoxide complexes, the difference was found in the reaction kinetics, which was dependent on the monomer used. The pseudo-first-order and zero-order dependences on the monomer concentration were observed for *ε*-CL and for LA polymerization, respectively [30]. The titanium complexes bearing two *o*-vanillin-derived phenoxy-imine ligands were shown to adopt three coordination modes, each featuring six-membered N−O chelation and/or five-membered O−O chelation, depending on the imine nitrogen substituent steric bulk. The ROP of lactones was the most effective for complexes showing five-membered O−O chelation by both the ligands [31]. Other titanium complexes supported by two bidentate [ON]-type ligands were investigated in the polymerization of *ε*-CL and substituted *ε*-caprolactones [32]. The salicylbenzothiazole titanium complexes were found to be more active than their salicylbenzoxazole counterparts. A study by Lai et al. revealed that the presence of electron-withdrawing groups in the pyridine ring or steric bulky groups in hydroxymethyl groups of titanium complexes bearing 2,6-bis(*o*-hydroxyalkyl)pyridine ligands reduced the catalytic activity of those complexes in *ε*-CL and LA polymerizations [33]. Homoleptic titanium complexes bearing unsymmetrical sulfonamide-supported salalen ligands showed good catalytic activity in the ROP of both the *ε*-CL and LA. That polymerization yielded high molar mass polymers with narrow molar mass distributions and the PLA produced from *rac*-lactide (*rac*-LA) was atactic [3]. An atactic polymer was also formed by homoleptic titanium complexes bearing tridentate [ONO]-type Schiff base ligands [34]. In contrast, heterotactic PLA with high stereoselectivity or an atactic one can be produced by the titanium complexes with tetradentate [NOOO]-type ligands, depending on the substituents on the ligand [35]. The stereoselective polymerization of *rac*-lactide was observed for the titanium complexes containing the tetradentate unsymmetric tertiary amine ligand [36]. The tetradentate non-symmetric amine bis(phenolate) titanium complexes offered good catalytic activity and they allowed the controlled polymerization of lactide monomers under melt conditions. Their activities were dependent on the ligand substitution pattern and increased for electron-withdrawing substituents [37]. In contrast, the catalytic activity of the bimetallic salen-type titanium complexes reported by Duan et al. decreased after the introduction of the *ε*-CL atoms on the ligands [38]. On the other hand, those complexes exhibited higher polymerization activity and better molar mass control of PLAs than their monometallic counterparts. The air- and moisture-stable Ti amino acid-derived amine bis(phenolate) complexes, which were obtained as polymetallic aggregates of different nuclearity, showed low activity in the ROP of *rac*-lactide. However, smaller binuclear Ti aggregates exhibited relatively better efficiency in that process compared to bigger Ti tetramers [39]. Upitak et al. compared the literature data on a five-coordinated titanium complex with a tridentate bis(phenolate)-amine ligand and six-coordinated analogs with tetradentate ligands. They found out that a lower coordination number around the titanium center and fewer sterically encumbered ligands provided more active and better-controlled initiators for *ε*-CL polymerization [40]. The titanium complexes bearing [ONO]-tridentate Schiff bases were also shown to be more active for both LA and *ε*-CL polymerization processes than the titanium complexes bearing other multiple-dentate ligands. This possibly results from the reduced space, which is occupied by the ligand, and from the increased monomer coordination around the metal center [29]. The presented results do not clearly reveal the influence of the ligand structures on the performance of the titanium complexes in the ROP of cyclic monomers. In addition, it is hard to directly compare the literature data obtained by various research groups because of different reaction conditions (temperature, presence of solvent or solvent-free conditions) and because of uncertainties regarding the number of initiating groups. Therefore, in order to gain insight into the effects of particular elements of the ligand structures on the catalytic behavior of complexes, we synthesized new tridentate ligands with a different steric bulk of phenolate substituents and different types of nitrogen donor groups.

Despite numerous reports that describe the behavior of titanium complexes in the ROP, there have been published only very few papers on vanadium complexes bearing ligands with O and N donor atoms, e.g., bi- and tridentate phenoxy-imine, tetradentate [ONNO]-type, and ligands derived from potentially tridentate 6-bis(o-hydroxyaryl)pyridines [41,42,43]. All of them were shown to promote the polymerization of *ε*-caprolactone but they turned out to be significantly less effective than titanium complexes with multidentate ligands. However, there is no information available about the use of such complexes in the polymerization of lactide monomers.

In this contribution, we report the synthesis and characterization of a series of titanium isopropoxide complexes, which contain tridentate [ONO]-type ligands, and we present the catalytic behavior of those complexes in the ROP of cyclic esters (*rac/L*-lactide and *ε*-caprolactone) under solvent-free conditions. Our aim was to explore the impact of the type of nitrogen donor group (imine or amine) and the effect of the presence of a substituent in the phenolate ring (H or *t*Bu) on the complex performance. Furthermore, vanadium complexes with tridentate phenoxy-imine ligands were prepared and their catalytic performance was compared with that of titanium counterparts under similar conditions.

## 2. Results and Discussion

### 2.1. Synthesis of Complexes

**Scheme 1:**
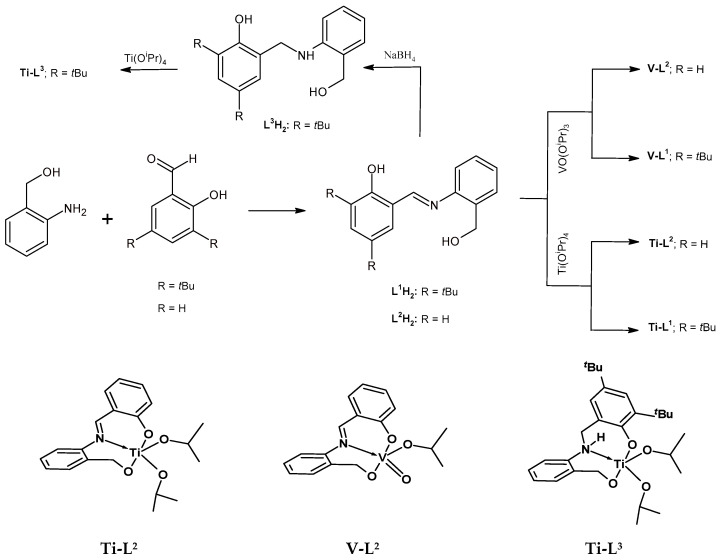
Synthesis of [ONO]-type tridentate proligands and corresponding titanium and vanadium complexes and proposed structures of new titanium (**Ti-L^2,3^**) and vanadium (**V-L^2^**) complexes.

**Figure 1:**
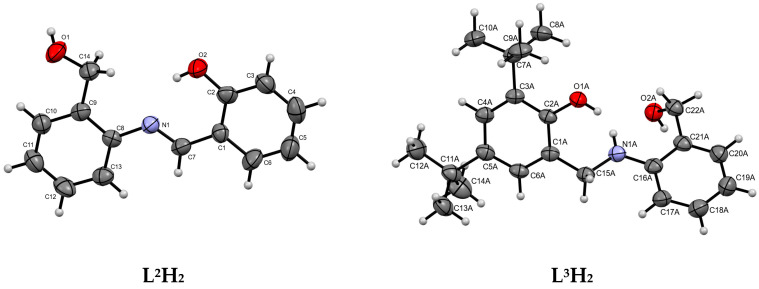
Molecular structures of compound **L^2^H_2_** and **L^3^H_2_**. Displacement ellipsoids are drawn at the 50% probability level. In **L^3^H_2_**, the disordered molecules have been removed from the drawings for clarity.

The synthesis and characterization of the phenoxy-imine proligand, **L^1^H_2_**, as well as the corresponding titanium (**Ti-L^1^**) and vanadium (**V-L^1^**) complexes, were reported in our previous communication [41]. In this work, we synthesized two novel [ONO]-type proligands and corresponding complexes. The new proligands differed from previously known **L^1^H_2_** by the absence of *t*Bu substituents in the phenolate ring (**L^2^H_2_**) or the presence of an amine donor instead of an imine one (**L^3^H_2_**). The phenoxy-imine proligand **L^2^H_2_** was synthesized by condensing 2-aminobenzyl alcohol and salicylaldehyde in methanol. The phenoxy-amine proligand, **L^3^H_2_**, was synthesized by the reduction of **L^1^H_2_** with sodium borohydride (Scheme 1). The phenoxy-imine compound was formed as a yellow powder with a good yield (81%); whereas, the phenoxy-amine one was obtained as a white powder with moderate yield (58%). The single X-ray structures of **L^2,3^H_2_** proligands were determined at 293 K and at 100 K for **L^2^H_2_** and **L^3^H_2_**, respectively (Figure 1). The result indicated that both proligands were crystallized in the monoclinic crystal system with the space group P2_1_/n (phenoxy-imine) and P2_1_/c (phenoxy-amine). The crystal and refinement data for the studied compounds are provided in Table S1 and selected geometric parameters are provided in Table S2. There is one molecule in the asymmetric part of the unit cell of the **L^2^H_2_** compound. In the crystal, the molecules are connected by strong O-H⋯O hydrogen bonds and they form layers parallel to (001). In addition, there is one intramolecular N-H⋯O hydrogen bond. There are two molecules in the asymmetric part of the unit cell of the **L^3^H_2_** compound. One of them is disordered. Two molecules in the unit cell are connected by strong O-H⋯O and N-H⋯O hydrogen bonds. The disordered fragments have been removed from the drawings to show more clearly the proligand structure.

**Figure 2:**
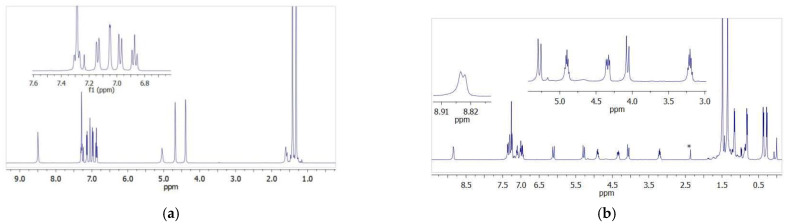
^1^H NMR spectra: (**a**) spectrum of the phenoxy-amine proligand; (**b**) spectrum of the corresponding titanium complex (*-toluene).

The stoichiometric reaction of the **L^2,3^H_2_** ligand precursors with one equivalent of titanium(IV) tetraisopropoxide in methylene chloride produced **Ti-L^2^** and **Ti-L^3^** titanium complexes, which were then isolated as a pale yellow powder and a yellow powder in quantitative yields. Vanadium(V) oxytriisopropoxide was reacted with the **L^2^H_2_** proligand to provide a dark brown powder (complex **V-L^2^**) in high yield. All new compounds were characterized by ^1^H and ^13^C NMR spectroscopy, as well as by the FTIR method. The exemplary spectra are presented in Figure 2 while other NMR and FTIR spectra have been included in the supporting materials (Figures S1–S10). The formation of the **Ti-L^2^** and **V-L^2^** phenoxy-imine complexes was confirmed by the disappearance of the proton signal of the Ar-O**H** hydroxyl group, which could be observed at 13.09 ppm in the spectrum of **L^2^H_2_**, and by the new resonance signals that appeared, which could be assigned to the methyl and methine protons of the isopropoxy group bounded to the metal center. The methylene protons of the Ar-C**H**_2_-O unit are inequivalent in complexes; thus, they gave rise to two doublets at 4.60 and 5.14 ppm for **Ti-L^2^** and 5.52 and 5.81 ppm for **V-L^2^**, compared to one doublet at 4.89 for the free proligand. In the spectrum of the **Ti-L^3^** complex, Ar-C**H**_2_-N and Ar-C**H**_2_-O methylene protons appear as doublets at 4.06 (1H), 5.24 (1H), and 6.11 (1H) ppm and one doublet of a doublet at 4.34 ppm (1H) while these signals for the proligand were observed as singlets at 4.36 and 4.65 ppm. In addition, the signal for the amine proton was shifted upon complexation from 8.52 (singlet) to 8.84 ppm (doublet). The signals for the methine and methylene protons of the isopropoxy groups appeared as multiplets at 3.20 and 4.90 ppm and as four doublets at 0.27, 0.37, 0.81, and 1.16 ppm.

When the FTIR spectra of the **L^2^H_2_** phenoxy-imine proligand and the corresponding complexes (Figure S1) are compared, it can be seen that the band at 3150 cm^−1^, which is assigned to ν(O-H), disappears upon complexation and the band at 1617 cm^−1^, due to the C=N stretching vibrations, is shifted to lower wavenumbers in the spectra of complexes (1608 cm^−1^ for **Ti-L^2^** and 1614 cm^−1^ for **V-L^2^**). The FTIR spectrum of the phenoxy-amine proligand (Figure S2) shows the bands at 3327 cm^−1^ and 3520 cm^−1^ corresponding to the hydroxyl groups stretching vibrations. Those bands are absent in the spectrum of **Ti-L^3^**, which confirms the deprotonation of the –OH groups and the formation of the Ti-O bonds. The formation of each of the synthesized complexes is further supported by the new bands appearing in the 650–450 cm^−1^ region due to M–O and M←N vibrations. The titanium complexes also exhibited the new bands in the region of 994–1038 cm^−1^, which can be assigned to ν(C-O) for the metal isopropoxy group. The values within the scope of vibrations of 957–978 cm^−1^ in the oxovanadium(v) complex are due to ν(C-O) and ν(V=O) [44,45]. The proposed structures of the new complexes (**Ti-L^2,3^**, **V-L^2^**) are provided in Scheme 1. However, on the basis of the data available, we cannot exclude that the complexes have dimeric forms like titanium complex bearing L^1^ ligand [41].

### 2.2. Polymerization of Lactides

**Figure 3:**
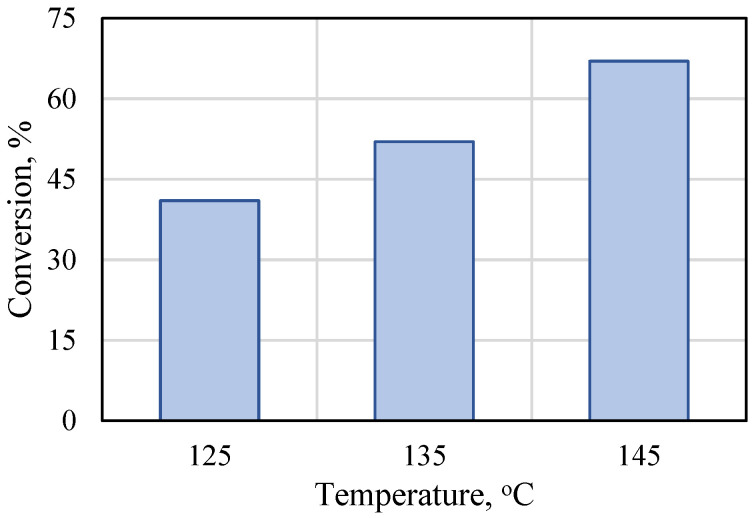
Effect of reaction temperature on the bulk polymerization of *rac*-lactide in the presence of Ti-L^2^ ([*rac*-LA]/[Ti] = 175, 60 min).

The effect of temperature on the bulk polymerization of *rac*-lactide was evaluated for the **Ti-L^2^** catalyst at the monomer-to-catalyst molar ratio and polymerization time fixed at 175 and 60 min, respectively. The conversion reached 44% at 125 °C and, as expected, the monomer conversion increased with the increasing process temperature up to 67% when the reaction was conducted at 145 °C (Figure 3). For comparative purposes, Table S6 summarizes the results of the polymerization of lactide with other titanium catalysts. Further tests were therefore carried out at 145 °C.

**Figure 4:**
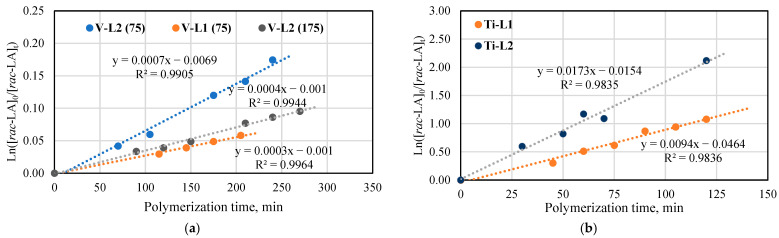
Plot of ln([LA]_0_/[LA]_t_) vs. time for *rac*-lactide polymerization: (**a**) Polymerization in the presence of **V-L^1−2^** at the molar ratio of [*rac*-LA]/[V] equal to 75 and 175; (**b**) Polymerization in the presence of **Ti-L^1−2^** at the molar ratio of [*rac*-LA]/[Ti] equal to 175 (**b**) in the bulk at 145 °C.

For the kinetic studies, the ring-opening polymerizations of *rac*-lactide with the use of the phenoxy-imine titanium (**Ti-L^1−2^**) and vanadium (**V-L^1−2^**) complexes were carried out at the monomer/metal molar ratio equal to 175 or 75 in the bulk at 145 °C. The results presented in Figure 4 show the linear relationships between ln([*rac*-LA]_0_/[*rac*-LA]_t_) and time for polymerizations catalyzed by all phenoxy-imine complexes, which is indicative of the first-order dependence on monomer concentration. The apparent rate constants (k_app_), as calculated from the slopes of the lines, are much higher for the titanium catalysts (k_app_ = 0.0094 min^−1^ for **Ti-L^1^** and 0.0173 min^−1^ for **Ti-L^2^**) than for the vanadium ones (k_app_ = 0.0003 min^−1^ for **V-L^1^** and 0.0004 ([*rac*-LA]/[V] = 175) and 0.0007 min^−1^ ([*rac*-LA]/[V] = 75) for **V-L^2^**). Moreover, the results suggest that the polymerization rate is approximately twice as high in the presence of complexes with ligands without *tert*-butyl substituents on the phenolate ring as compared to their counterparts with these substituents. It can be explained by the higher steric volume of *t*Bu substituents, which makes more difficult the access of the monomer to the metal center. The data collected for polymerization with **V-L^2^** testify that the increase in the monomer-to-catalyst molar ratio from 75 to 175 reduces the monomer conversion level and the k_app_ value.

**Figure 5:**
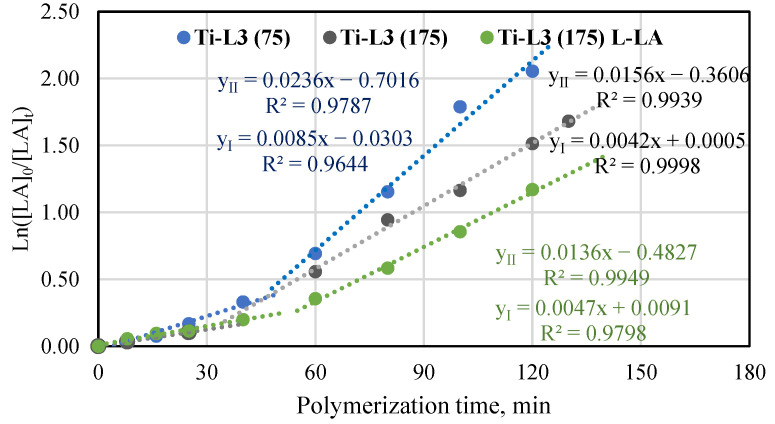
Plot of ln([LA]_0_/[LA]_t_) vs. time for *rac*-lactide and *L*-lactide polymerization in the presence of **Ti-L^3^** at the molar ratio of [LA]/[Ti] equal to 75 and 175 in the bulk at 145 °C.

For comparison, the **Ti-L^3^** phenoxy-amine complex, an analogue of the **Ti-L^1^** phenoxy-imine complex, was also tested in *rac*-lactide polymerization under the same conditions. As can be seen in Figure 5, the semilogarithmic relationship for polymerization in the presence of the phenoxy-amine complex is different from the one obtained for the phenoxy-imine complex. Polymerization proceeds with a slow induction period followed by polymerization at a higher rate. A curved line was divided into two essentially linear stages with different slopes, which allowed us to calculate the k_app_ for each stage. Thus, the polymerization process is the first-order reaction from the viewpoint of the monomer concentration in its first and second stages; however, the apparent polymerization constant is higher in the second stage. In order to confirm that relation, the polymerization reaction of *L*-lactide in the same polymerization conditions and the additional polymerization of *rac*-lactide at a lower monomer-to-titanium molar ratio were conducted (Figure 5). Both these polymerizations also proceed in two linear stages. Such a phenomenon was previously observed for *L*-lactide polymerization in the presence of the yttrium complex [Y(pdtbp){N(SiHMe_2_)_2_}(THF)] (pdtbp = 1,5-dithiapentanediyl-bis{4,6-di-*tert*-butylphenolato}) [46]. The results also confirmed that *rac*-lactide was converted into a polymer faster than *L*-lactide was. However, the differences were minor, as indicated by the similar k_app_ values for the first stage and slightly higher k_app_ values for the second stage (0.0156 min^−1^ and 0.0136 min^−1^, respectively). Comparison of the conversion results for T-L^1^ and **Ti-L^3^** in *rac*-LA polymerization after 120 min (Table S5) showed higher overall activity of the complex with the amine group (78% vs. 66%). 

**Figure 6:**
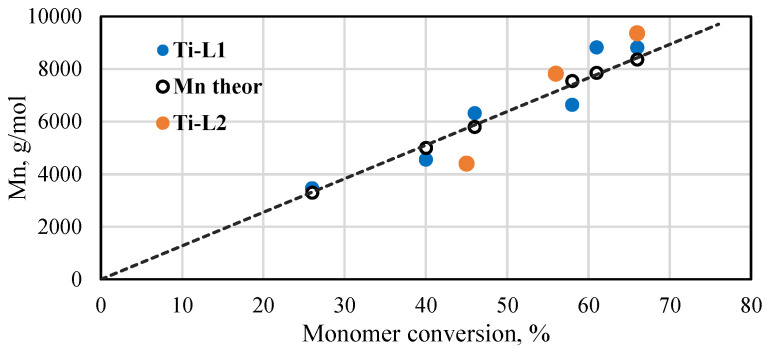
Plot of M_n_ of poly(*rac*-lactides) synthesized with the phenoxy-imine titanium complexes as a function of monomer conversion ([*rac*-LA]/[Ti] = 175, 145 °C, M_n_ of polymers calculated from NMR spectra).

The molar masses of PLAs were determined by ^1^H NMR spectroscopy. It was difficult to determine the exact molar masses of PLAs produced with the vanadium complexes. The analysis was subject to measurement error due to the small amount of the product in the reaction mixture and the lower quality of the spectrum. However, several results that were obtained (Table S3) indicate that the molar masses of resulting products are very low and their values are close to the theoretical ones (assuming that one isopropoxy group takes part in the reaction). The results for polymers synthesized with the **Ti-L^1−2^** titanium complexes are presented in Figure 6. A linear increase in the M_n_ values can be seen with respect to the monomer conversion. Additionally, the experimental molar masses are quite close to their theoretical values, which were calculated taking into account that both the isopropoxide groups that are present in the titanium complexes will initiate the polymerization reaction. The molecular weight distributions (M_w_/M_n_) of the exemplary samples determined by the GPC method (Figure S11) are relatively narrow, equal to 1.26 and 1.39 for PLA produced with **Ti-L^1^** and **Ti-L^2^**, respectively. These results demonstrate that lactide polymerization in the presence of phenoxy-imine complexes is rather well-controlled.

**Figure 7:**
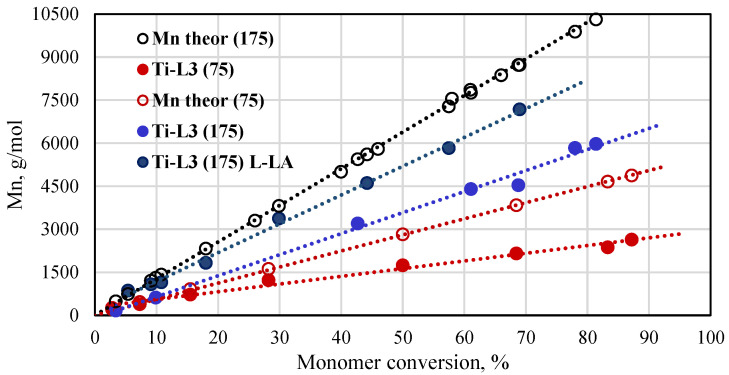
Plot of M_n_ of poly(*rac*-lactides) and poly(*L*-lactides) synthesized with the phenoxy-amine titanium complex as a function of conversion ([*rac*-LA]/[Ti] = 75 or 175, [*L*-LA]/[Ti] = 175, 145 °C, M_n_ of polymers calculated from NMR spectra).

A plot of theoretical and experimental molar mass vs conversion for the polymerization reactions of *L*-lactide (*L*-LA) and *rac*-LA in the presence of the **Ti-L^3^** phenoxy-amine titanium complex is presented in Figure 7. The molar masses of the resulting polymers increased linearly with the increase in the monomer conversion. The experimental molar masses for poly(*rac*-lactide), independently from the monomer-to-catalyst molar ratio used, are lower than the theoretical values. The *M_n NMR_* values obtained for poly(*L*-lactide) show better compliance with the theoretical ones but, still, they are lower than expected. Similar results, i.e., better fit of experimental and theoretical molar masses for poly(*L*-lactide) than for poly(*rac*-lactide), are reported in the literature for polymers that were produced with the use of titanium complexes based on aminodiol ligands [47].

**Figure 8:**
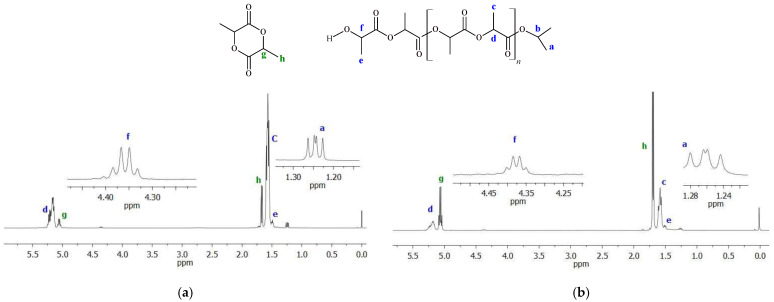
Exemplary ^1^H NMR spectra of poly(*rac*-lactides) (in the reaction mixture): (**a**) PLA obtained with Ti-L^2^ after 120 min; (**b**) PLA produced with **Ti-L^3^** after 60 min (conditions: 145 °C, [LA]/[Ti] = 175).

The ^1^H NMR analysis (exemplary spectra are shown in Figure 8) of end groups in PLAs and PLLAs obtained with the use of the investigated titanium complexes revealed the presence of hydroxyl and isopropyl ester end groups, which suggests that the reaction was initiated through the insertion of lactide monomers into the M-O bond via the coordination insertion mechanism [47,48]. The ^13^C NMR spectra of PLAs (Figure S12) derived from *rac*-LA show several lines in the carbonyl region (169.16, 169.34, 169.38, 169.43, 169.58, and 169.63 ppm), which are specific for poly(*rac*-lactide); these are assigned to the *isisi*, *isiii*, *iisii/sisii/sisis*, *iiisi/siiis*, and *iiiii/iiiis/siiii* hexads. The resonances at 69.01 and 69.19 ppm, which correspond to the *iii*/*iis*/*sii*/*sis* and *isi* sequences, are present in the methine region. The good agreement of intensities of these signals with theoretical values (75:25) suggests that the sequence distribution follows pair-addition Bernoullian statistics and that the transesterification reaction is negligible in polymerization catalyzed by titanium complexes [49,50,51]. The selected polymer products were analyzed by the DSC method (Figure S13). The thermograms of investigated poly(*rac*-lactides) show only the presence of the glass transition temperatures For example, the *T_g_* of PLA synthesized with **Ti-L^1^** and **Ti-L^2^** fell in the ranges of 40.8–48.4 °C and 44.5–51.1 °C, depending on the polymer molar mass.

### 2.3. Polymerization of ε-Caprolactone 

**Figure 9:**
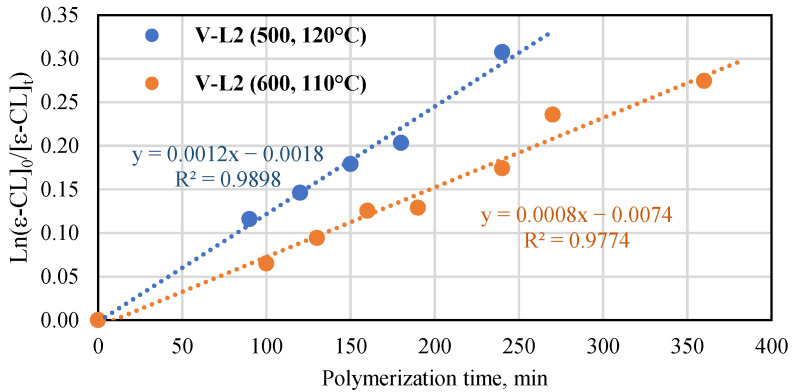
Plot of ln([*ε*-CL]_0_/[*ε*-CL]_t_) vs. time for *ε*-caprolactone polymerization in the presence of **V-L^2^** in the bulk ([*ε*-CL]/[V] = 500, 120 °C and [*ε*-Cl]/[V] = 600, 110 °C).

The performance of the vanadium and titanium complexes with phenoxy-imine ligands (**Ti-L^1^** and **V-L^1^**) towards the ROP of *ε*-caprolactone was the subject of our previous research [41]. Therefore, we studied the polymerization reactions carried out with the use of **Ti-L^2^** and **V-L^2^** as catalysts. The monomer-to-metal molar ratio was fixed at 600:1 (Table S4). The titanium complex performed better than the vanadium complex. The monomer conversion and M_n NMR_ of PCL after 13 min in the reaction with the use of **Ti-L^2^** were 46.5% and 13,500 g/mol at 110 °C, 39.5% and 10,800 g/mol at 100 °C, and 15.4% and 4600 g/mol at 90 °C. Extending the polymerization time to 25 min at 90 °C resulted in an increase in conversion and molecular mass to 35.1% and 10,200 g/mol. The theoretical number-average molar masses calculated for two polymer chains growing on one titanium center were equal to 15,700, 13,600, 5030 g/mol, and 12,100 g/mol, respectively. Hence, the experimental values of M_n NMR_ were quite close to the theoretical ones. Moreover, the results of the GPC analysis included in Table S4 are very similar to the results obtained by the NMR method. The investigated polycaprolactones are also characterized by a narrow molar mass distribution (M_w_/M_n_ = 1.22–1.33). However, it should be noted that at higher temperatures (at 100 and 110 °C), in addition to the polymer, a fraction of low molecular weight oligomer with M_n_ of approximately 400 g/mol is formed. The polymerization reaction catalyzed by **V-L^2^** at 110 °C provided the monomer conversion of 9% and M_n NMR_ of 5500 g/mol after 130 min. The theoretical M_n_ calculated for one polymer chain growing on one vanadium center was equal to 6140 g/mol. The data of conversion versus time were collected for the vanadium complex at two sets of conditions: 110 °C, [*ε*-CL]/[V] = 600:1 and 120 °C, [*ε*-CL]/[V] = 500:1 (Figure 9). As can be seen, the first-order kinetic plots for ln[ε-CL]_0_/[*ε*-CL]_t_ vs. time were obtained in both cases. The k_app_ values were equal to 0.0008 and 0.0012 min^−1^ for the processes conducted at 110 °C and at 120 °C, respectively. The results presented in Table S4 for polymerization at the lower temperature revealed similar values of molar masses as determined by the ^1^H NMR method and the theoretical ones, which is indicative of a well-controlled process. However, the molar masses of polymers synthesized at 120 °C were different from the expected values. That difference can result from side reactions at elevated temperatures [52].

**Figure 10:**
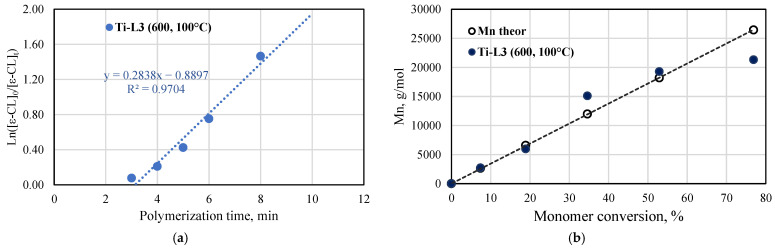
*ε*-caprolactone polymerization in the presence of **Ti-L^3^**: (**a**) Plot of ln([*ε*-CL]_0_/[*ε*-CL]_t_) vs. reaction time; (**b**) Plot of M_n_ of produced poly(*ε*-caprolactones) as a function of conversion (conditions: [*ε*-CL]/[Ti] = 600, 100 °C; M_n_ of polymers calculated from NMR spectra).

The kinetic studies of ε-caprolactone polymerization catalyzed by the titanium complex bearing phenoxy-amine ligand were carried out at 100 °C and at [ε-CL]/[V] = 600:1. The semilogarithmic plot of ln[ε-CL]_0_/[ε-CL]_t_ vs. time and the number of average molar masses M_n_ for the produced PCLs, as determined by ^1^H NMR spectroscopy, are shown in Figure 10. The kinetics indicated that the polymerization rate is of first-order with respect to the monomer concentration (k_app_ = 0.2838 min^−1^); thus, the process is consistent with the coordination insertion mechanism. Furthermore, a short induction period is observed before the polymerization is initiated. Such a phenomenon was observed before for the ε-CL polymerization in which titanium complexes were involved, among others, the complexes with pyrrolylaldiminate ligands [40]. The polymerization is well controlled, which is confirmed by the molar mass values. The experimental M_n_ values increase linearly with the monomer conversion and they are close to the theoretical values. For selected polycaprolactone samples, the molar masses and their distributions were determined with the use of the GPC method. The M_n_, M_w_, and dispersity values are provided in Figure S14. The tested samples showed a narrow molar mass distribution (M_w_/M_n_ = 1.22–1.99) and good agreement between M_n GPC_ and M_n NMR_ values.

**Figure 11:**
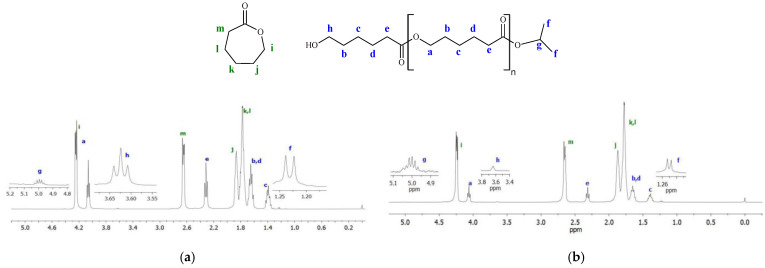
Exemplary ^1^H NMR spectra of PCLs (in the reaction mixture): (**a**) PCL obtained with **Ti-L^3^** after 5 min at 100 °C; (**b**) PCL produced with **Ti-L^2^** after 13 min at 90 °C ([*ε*-CL]/[Ti] = 600).

The ^1^H NMR spectra (Figure 11) show that the poly(*ε*-caprolactone) chains produced by the investigated titanium complexes are terminated with the isopropyl ester group (peaks *f* and *g* corresponding to methyl and methine protons) on one end and with the methylene hydroxyl group (peak *h*) on the other end. This indicates that *ε*-CL polymerization, similarly to LA polymerization, proceeds according to the coordination insertion mechanism. Such a mechanism is typical for the ROP of cyclic esters in the presence of isopropoxy titanium complexes supported by multidentate ligands [29,31,53]. The DSC thermograms recorded during the second heating of PCLs synthesized with vanadium and titanium complexes are shown in Figures S15 and S16, respectively. Only the characteristic endothermic peak was present in each thermogram within the tested range (10–150 °C). The melting points of PCLs were similar and they ranged from 54.3 to 57.2 °C; crystallinities of PCLs were in the range of 44.7–59.7%.

## 3. Materials and Methods

### 3.1. Materials

Argon (grade 5.0, Linde Gas), Ti(O*^i^*Pr)_4_ (99.999%, Sigma-Aldrich, St. Louis, MO, USA), VO(O*^i^*Pr)_3_ (TCI), NaBH_4_ (98%, Sigma-Aldrich), 2-aminobenzyl alcohol (98%, Sigma-Aldrich), and salicylaldehyde (99%, Acros Organic, Geel, Belgium) were used as received. CDCl_3_ (99.5 atom % D, Deutero GmbH, Kastellaun, Germany, 99.8 atom % D, 0.03% TMS), CH_2_Cl_2_ (Chempur, Piekary Śląskie, Poland, analytical grade), and *ε*-caprolactone (99%, Alfa Aesar, Haverhill, MA, USA) were dried over molecular sieves. *Rac*-lactide (99%, Acros Organic, Geel, Belgium) and *L*-lactide (99%, Aldrich) were purified by vacuum sublimation at 85°C. Hexane (Linegal Chemicals, Blizne Łaszczyńskiego, Poland) and toluene (FP, AnalaR NORMAPUR, VWR, Radnor, PA, USA) were refluxed over sodium and sodium/benzophenone, respectively, and distilled under a nitrogen atmosphere prior to use.

### 3.2. Methods 

All manipulations of air- and/or moisture-sensitive materials were performed under an argon atmosphere using a dual vacuum/argon line, glove box, and Schlenk-type glassware. The infrared spectra were recorded with the Nicole Nexus 2002 FTIR spectrometer in the range of 4000 to 400 cm^−1^ with a 2 cm^−1^ resolution. The samples of proligands and complexes were prepared in Nujol and the polymer samples were prepared as KBr tablets/pills. Additionally, ^1^H and ^13^C NMR spectra were taken on the Bruker Ultrashield 400 spectrometer at room temperature using CDCl_3_ as a solvent. Chemical shifts were reported in ppm and were referenced to the solvent residue peak or TMS peak. Differential scanning calorimetry (DSC) thermograms were acquired with a 2010 DSC calorimeter from TA Instruments at the heating rate of 10 °C/min. The heating-cooling-heating cycles were performed and the glass transition (T_g_), melting temperatures (T_m_), and crystallinities (χ) of polymers were obtained from the second heating. The poly(*ε*-caprolactone) crystallinity was calculated assuming that the enthalpy of melting of a completely crystalline polymer is equal to 139.5 J/g [54]. Molar masses and dispersities of polymers were measured by gel permeation chromatography (GPC) using Agilent 1260 Infinity equipped with the Phenogel 10 µm Linear(2) 300 × 7.8 mm column. Analyses were made at 35 °C using THF as a solvent under a flow rate of 1.0 mL/min. Polystyrene standards with molar masses ranging from 1000 to 3,500,000 g/mol were used for calibration. Obtained M_n_ values were multiplied by 0.58 or 0.56 (correction coefficient for polylactide and polycaprolactone, respectively) to correlate them to actual values [55]. The single crystal of the compound **L^2^H_2_** was collected on a Kuma KM4 diffractometer equipped with the Eos CCD detector (graphite monochromatic, MoK*α* radiation, λ = 0.71073 Å) at room temperature. The single crystal of the compound **L^3^H_2_** was collected on a Rigaku Oxford Diffraction XtaLAB SynergyR DW diffractometer equipped with a HyPix ARC 150° Hybrid Photon Counting (HPC) detector using CuK*α* (λ = 1.54184 Å) at 100 K. The corrections to the Lorentz and polarization factors were applied to the reflection intensities [56,57]. Data were processed using the CrysAlisPro Software System (Version 1.171.37.57). The structures were solved by direct methods using SHELXS and refined by full-matrix least-squares methods based on F^2^ using SHELXL [58,59]. The hydrogen atoms were determined from the geometric concepts and refined in a riding model with isotropic temperature factors of 1.2 times the Ueq value of the parent atom. All non-hydrogen atoms were located by difference Fourier synthesis and refined by the least squares method in the full-matrix anisotropic approximation. The crystallographic data for compounds and details of the X-ray experiment are collected in the Supplementary Information Tables. The structure drawings in ESI were prepared by using the Mercury program [60]. The coordinates of atoms and other parameters for structures were deposited with the Cambridge Crystallographic Data Centre: 2298801 for 1, 2298802 for 2, 12 Union Road, Cambridge CB2 1EZ, UK (Fax,_44-(1223)336-033, E-mail deposit@ccdc.cam.ac.uk).

### 3.3. Synthesis of Ligand Precursors

Additionally, 2-[{2-(hydroxymethyl)phenylimino}-methyl]-4,6-di-*tert*-butylphenol (**L^1^H_2_**) was synthesized according to the procedure described in our previous report [41]. 

2-[{2-(hydroxymethyl)phenylimino}-methyl]-phenol (**L^2^H_2_**). In addition, 2-Aminobenzyl alcohol (2.48 g, 0.02 mol) and salicylaldehyde (2.10 mL, 0.02 mol), in 60 mL of methanol, were refluxed for 5 h. Then, the solvent was evaporated and the residue (3.84 g) was purified chromatographically. **L^2^H_2_** (3.67 g, 81%) was obtained as yellow powder. ^1^H NMR (400 MHz, CDCl_3_, ppm): 1.75 (t, 1H, Ar-CH_2_-O**H**), 4.87 (d, 2H, Ar-C**H_2_**-OH), 6.99–6.93 (td, 1H, Ar-H), 7.04 (d, 1H, Ar-H), 7.15 (dd, 1H, Ar-H), 7.32 (td, 1H, Ar-H), 7.46–7.35 (m, 3H, Ar-H), 7.53 (d, 1H, Ar-H); 8.62 (s, 1H, HC=N); 13.09 (s, 1H, Ar-OH). ^13^C NMR (100.6 MHz, CDCl_3_): 163.38, 161.18, 146.86, 134.54, 133.61, 132.55, 129.07, 128.50, 127.26, 119.37, 118.19, 117.45, 62.09. FTIR (Nujol, cm^−1^): 3139 ν(O-H), 1617 ν(C=N). 

3-[{2-(hydroxymethyl)phenylamino}-methyl]-4,6-di-*tert*-butylophenol (**L^3^H_2_**). Additionally, 2-[{2-(hydroxymethyl)phenylimino}methyl]-4,6-ditertbutylphenol (7.11 g, 0.02 mol) was dissolved in 100 mL methanol and then sodium borohydride (3.0 g) was added. The reaction proceeded until solution discoloration. upon the addition of water (80 mL), a white precipitate appeared, which was filtered off. The crude product was first crystallized from the ethanol/water solution and then the obtained crystals (6.16 g) were recrystallized from the diethyl ether/hexane mixture. In total, 4.0 g of **L^3^H_2_** were obtained as white crystals (58%). ^1^H NMR (400 MHz, CDCl_3_): δ 1.34 (s, 9H, *t*Bu), 1.44 (s, 9H, *t*Bu), 4.42 (s, 2H, Ar-C**H_2_**-NH), 4.71 (s, 2H, Ar-C**H_2_**-OH), 5.07 (s, 1H, Ar-CH_2_-O**H**), 6.90 (t, 1H, Ar-H), 7.00 (d, 1H, Ar-H), 7.07 (d, 1H, Ar-H), 7.16 (d, 1H, Ar-H), 7.21–7.23 (2H, Ar-H); 8.52 (s, 1H, NH-Ar). ^13^C NMR (100.6 MHz, CDCl_3_): δ 153.59, 146.71, 141.65, 136.37, 129.84, 129.26, 127.38, 123.89, 123.73, 122.44, 120.04, 114.08, 64.47, 48.66, 35.10, 34.39, 31.82, 29.82. FTIR (Nujol, cm^−1^): 3327, 3520 ν(O-H), 3572 ν(N-H).

### 3.4. Synthesis of Complexes

Isopropoxy titanium and vanadium complexes with the phenoxy-imine ligand **L^1^** (**Ti-L^1^** and **V-L^1^**) were synthesized according to the procedure described in our previous report [41].

*Synthesis of **Ti-L^2^***. A solution of Ti(O*^i^*Pr)_4_ (0.41 mL, 1.38 mmol) in 5 mL of toluene was added dropwise into a solution of **L^2^H_2_** (313 mg, 1.38 mmol) in 15 mL of CH_2_Cl_2_ over 20 min. The reaction was carried out for 21 h at room temperature and then volatile materials were removed under reduced pressure. The residue was washed with hexane (8 mL) and its evaporation under a vacuum afforded the product as a pale yellow powder with a quantitative yield (98%). ^1^H NMR (400 MHz, CDCl_3_): δ 1.21–0.63 (12H, O-CH(CH_3_)_2_), 4.03 (m, 1H, O-CH(CH_3_)_2_); 4.60 (1H, Ar-CH_2_-O); 4.80–5.30 (overlapping, 2H, O-CH(CH_3_)_2_ and Ar-CH_2_-O), δ 7.08–6.80 (4H, Ar-H), 7.24–7.11 (2H, Ar-H), 7.52–7.41 (2H, Ar-H), 8.39 (s,1H, HC=N). ^13^C NMR (100.6 MHz, CDCl_3_): 25.49, 73.22, 77.36, 119.67, 121.32, 126.41, 127.26, 128.38, 134.23, 135.54, 138.07, 164.65. FTIR (Nujol, cm^−1^) 1608 ν(C=N).

*Synthesis of **V-L^2^***. A solution of VO(O^i^Pr)_3_ (0.36 mL, 1.52 mmol) in 10 mL of CH_2_Cl_2_ was added dropwise into a solution of **L^2^H_2_** (346 mg, 1.52 mmol) in 10 mL of CH_2_Cl_2_ over 26 min. The reaction was carried out for 22 h at room temperature and then volatile materials were removed under reduced pressure. The residue was washed with toluene (2 mL) and hexane (4 mL) and their evaporation under a vacuum afforded the product as a dark brown powder with a quantitative yield (99%). ^1^H NMR (400 MHz, CDCl_3_): two set of signals in the ratio of 4:1. Main isomer: 1.48 (two overlapping d, 6H, O-CH(CH_3_)_2_), 5.52 (d, 1H, Ar-CH_2_-), 5.81 (d, 1H, Ar-CH_2_-), 5.72 (m, 1H, O-CH(CH_3_)_2_), 6.95–7.60 (8H, Ar-H); 8.54 (s, 1H, CH=N). ^13^C NMR (100.6 MHz, CDCl_3_): δ 162.25, 148.16, 138.04, 137.22, 136.48, 136.13, 133.94, 129.19, 129.16, 128.78, 128.38, 127.85, 126.84, 126.50, 126.28, 121.72, 121.22, 120.99, 120.18, 119.74, 85.75, 64.61, 25.74, 25.53, 24.32, 24.17. FTIR (Nujol, cm^−1^) 1614 ν(C=N). 

*Synthesis of **Ti-L^3^***. A solution of Ti(O*^i^*Pr)_4_ (0.11 mL, 1.03 mmol) in 7 mL of CH_2_Cl_2_ was added dropwise into a solution of **L^2^H_2_** (352.8 mg, 1.03 mmol) in 12 mL of CH_2_Cl_2_ over 12 min. The reaction was carried out for 22 h at room temperature and then volatile materials were removed under reduced pressure. The residue was washed with hexane (7 mL) and its evaporation under a vacuum afforded the product as a pale yellow powder with a quantitative yield. ^1^H NMR (400 MHz, CDCl_3_): δ 0.27 (d, 3H, O-CH(CH_3_)_2_), 0.37 (d, 3H, O-CH(CH_3_)_2_), 0.81 (d, 3H, O-CH(CH_3_)_2_), 1.16 (d, 3H, O-CH(CH_3_)_2_), 1.34 (s, 9H, *t*Bu), 1.48 (s, 9H, *t*Bu), 3.20 (m, 1H, O-CH(CH_3_)_2_), 4.06 (d, 1H, Ar-CH_2_-), 4.34 (dd, 1 H, Ar-CH_2_-), 4.90 (m, 1H, O-CH(CH_3_)_2_), 5.24 (d, 1H, Ar-CH_2_-), 6.11 (d, 1H, Ar-CH_2_-), 7.04–6.92 (m, 2H, Ar-H), 7.10 (t, 1H, Ar-H), 7.24 (d, 1H, Ar-H), 7.30 (d, 1H, Ar-H), 7.36 (d, 1H, Ar-H), 8.84 (d, 1H, CH_2_-NH-Ar). ^13^C NMR (100.6 MHz, CDCl_3_): δ 162.28, 145.89, 139.99, 136.56, 134.16, 129.19, 128.38, 125.50, 125.36, 124.46, 123.00, 122.34, 117.15, 79.10, 77.36, 75.86, 73.88, 47.27, 35.27, 34.82, 34.68, 34.34, 31.96, 30.37, 27.07, 26.97, 25.47, 25.16, 24.98. FTIR (Nujol, cm^−1^) 3616 ν(N-H).

### 3.5. Polymerization of Cyclic Esters 

*Bulk polymerization of rac-lactide and L-lactide.* Typically, about 15 mg of a complex and the appropriate amount of lactide monomer, resulting from the assumed monomer/transition metal molar ratio, was put into a Schlenk tube in a glove box under argon. The tube was placed in the oil bath, which had been pre-heated to the polymerization temperature, as chosen for a test. An aliquot of the polymerization mixture was periodically or at the end of the reaction taken out from the flask and subjected to ^1^H NMR spectroscopy to determine the monomer conversion and average molar masses of products. The reaction was quenched with the use of methanol; the polymer was dissolved in a minimum amount of CH_2_Cl_2_ and precipitated in cold methanol. The polymer was separated by filtration (if precipitated), washed with methanol, and dried in vacuo at room temperature to constant weight. Monomer conversion was calculated from the relative intensity of the signals at *δ* 5.06 and 5.18 ppm due to the -OCH(Me)- in lactide monomer and polymer, respectively [61]. The molar mass (M_n NMR_) of polylactides was calculated from the ratio of the isopropoxy end group signal at 1.22 ppm and the backbone resonance -OCH(Me)- at 5.18 ppm [47]. The theoretical values of M_n_ (M_n theo_) were calculated from the formula: molar mass of the monomer × ([monomer]_0_/(n × [M]_0_)) × conversion yield + 60.1 (for end group), where M = Ti and n = 2 or M = V and n = 1 [62].

*Bulk polymerization of ε-caprolactone.* Typically, about 15–20 mg of a complex was put into a Schlenk tube in a glove box under argon and then the appropriate amount of *ε*-caprolactone resulting from the assumed monomer/transition metal molar ratio was added by syringe. The tube was placed in the oil bath, which had been pre-heated to the polymerization temperature, as chosen for a test. An aliquot of the polymerization mixture was periodically, or at the end of the reaction, taken out from the flask and subjected to ^1^H NMR spectroscopy to determine the monomer conversion and average molar masses of products. The reaction was quenched with the use of methanol and the polymer was extracted with chloroform and precipitated in methanol. The polymer was separated by filtration, washed with methanol, and dried in vacuo at room temperature to constant weight. Monomer conversion was calculated from the relative intensity of the signals at *δ* 4.25 and 4.06 ppm due to the -OCH_2_- in ε-CL and PCL, respectively [31]. The molar mass (M_n NMR_) of PCL was calculated from the ratio of the isopropoxy end group signal at 1.22 ppm and backbone methylene signal (-OCH_2_-) at 4.06 ppm [53]. The theoretical values of M_n_ (M_n theo_) were calculated from the formula: molar mass of the monomer × ([monomer]_0_/(n × [M]_0_)) × conversion yield + 60.1 (for end groups), where M = Ti and n = 2 or M = V and n = 1 [53,63,64].

## 4. Conclusions

The studied titanium and vanadium complexes were demonstrated to promote the ROP of cyclic esters. The polymerization of *rac*-lactide and *ε*-caprolactone, when mediated by complexes bearing tridentate phenoxy-imine ligands, is well controlled under appropriate polymerization conditions and the kinetic experiments show the first-order dependence on the monomer concentration. The performance of a complex was strongly influenced by the ligand structure and the central metal. The titanium complexes showed clearly higher levels of activity for both *rac*-lactide and *ε*-caprolactone polymerizations against their vanadium counterparts. The presence of substituents at the *ortho*- and *para*-positions on the phenoxy ring of complexes led to a slower polymerization rate when compared with the complexes without such substituents. Hence, the observed changes can be attributed to the steric hindrance caused by the bulky *tert*-butyl groups. In contrast, a two-stage relationship was noted between ln([LA]_0_/[LA]_t_) and the polymerization time for the phenoxy-amine titanium complex in *rac*- and *L*-lactide polymerization. For both stages, the polymerization was a first-order reaction for the monomer concentration, with the increased apparent polymerization constant in the second stage. In the case of *ε*-caprolactone polymerization, a plot of ln [*ε*-CL]_0_/[*ε*-CL]_t_ vs. time exhibited an induction period before a gradual linear increase in the rate.

## Data Availability

Data are contained within the article and supplementary materials.

## Supplementary Materials

The following supporting information can be downloaded at: https://www.mdpi.com/article/10.3390/molecules29010087/s1, Table S1: Summary of crystal and refinement data for **L^2^H_2_** and **L^3^H_2_** proligands; Table S2: Selected geometric parameters for compounds **L^2^H_2_** and **L^3^H_2_** (Å, º); Figure S1: FTIR spectra of phenoxy-imine proligand **L^2^H_2_** and corresponding titanium Ti-L^2^ and vanadium **V-L^2^** complexes; Figure S2: FTIR spectra of phenoxy-amine proligand **L^3^H_2_** and corresponding titanium **Ti-L^3^** complex; Figure S3: ^1^H NMR spectrum of phenoxy-imine proligand **L^2^H_2_**; Figure S4: ^13^C NMR spectrum of phenoxy-imine proligand **L^2^H_2_**; Figure S5: ^1^H NMR spectrum of phenoxy-amine proligand **L^3^H_2_**; Figure S6: ^13^C NMR spectrum of phenoxy-amine proligand **L^3^H_2_**; Figure S7: ^1^H NMR spectrum of phenoxy-imine complex **V-L^2^**; Figure S8: ^13^C NMR spectrum of phenoxy-imine complex **V-L^2^**; Figure S9: ^1^H NMR spectrum of phenoxy-amine complex **Ti-L^3^**; Figure S10: ^13^C NMR spectrum of phenoxy-amine complex **Ti-L^3^**; Table S3: M_n NMR_ and M_n theo_ for PLA synthesized with vanadium complex **V-L^2^**; Figure S11: GPC curves of PLA synthesized with complexes **Ti-L^1^** and **Ti-L^2^**; Figure S12: ^13^C NMR spectra of the PLA obtained in the presence of **Ti-L^2^** (a) and **Ti-L^1^** (b); Figure S13: DSC thermograms of PLA synthesized with complexes **Ti-L^2^** and **Ti-L^1^**; Table S4: Results of ring-opening polymerization of *ε*-caprolactone catalyzed by **Ti-L^2,3^** and **V-L^2^**; Figure S14: GPC curves of PCL synthesized with complex **Ti-L^3^**; Figure S15: DSC thermograms of PCL synthesized with complex **V-L^2^**; Figure S16: DSC thermograms of PCL synthesized with complexes Ti-L^2^ and **Ti-L^3^**; Table S5: Selected results of lactide polymerization with titanium and vanadium complexes; Table S6: Literature data on the polymerization of lactide in the presence of titanium complexes containing ligands with O and N donor atoms. References [3,29,30,31,33,34,35,36,37,38] are cited in the supplementary materials.
