# Supplementary material for: Titanium and Vanadium Complexes of Tridentate Phenoxy-Imine and Phenoxy-Amine Ligands and Their Application in the Ring-Opening Polymerization of Cyclic Esters
Source: Molecules. 2023 Dec 22;29(1):87. doi: 10.3390/molecules29010087 (PMC10779786; doi:10.3390/molecules29010087)
Supplement: Supplementary file 1 [file molecules-29-00087-s001.zip › molecules-2737667-supplementary.pdf]

Supplementary Materials

Titanium and Vanadium Complexes of Tridentate Phenoxy-Imine and Phenoxy-Amine Ligands and Their Application in the Ring -Opening Polymerization of Cyclic Esters

Marzena Białek[1,*], Alicja Klimasińska[1], Grzegorz Spaleniak[1], Błażej Dziuk[2]

[1]*Institute of Chemistry, University of Opole, Oleska 48, 45-052, Opole, Poland*

[2]*Faculty of Chemistry, Wrocław University of Science and Technology,* Wybrzeże Wyspiańskiego 27, 50370 Wrocław, Poland

[*] Correspondence: marzena.bialek@uni.opole.pl

Table of contents	
Summary of crystal and refinement data for L^2H_2 and L^3H_2 proligands	**Table S1**
Selected geometric parameters for compounds L^2H_2 and L^3H_2 (Å, º)	**Table S2**
FTIR spectra of phenoxy-imine proligand L^2H_2 and corresponding titanium Ti-L^2 and vanadium V-L^2 complexes	**Figure S1**
FTIR spectra of phenoxy-amine proligand L^3H_2 and corresponding titanium Ti-L^3 complex	**Figure S2**
^{1}H NMR spectrum of phenoxy-imine proligand L^2H_2	**Figure S3**
^{13}C NMR spectrum of phenoxy-imine proligand L^2H_2	**Figure S4**
^{1}H NMR spectrum of phenoxy-amine proligand L^3H_2	**Figure S5**
^{13}C NMR spectrum of phenoxy-amine proligand L^3H_2	**Figure S6**
^{1}H NMR spectrum of phenoxy-imine complex V-L^2	**Figure S7**
^{13}C NMR spectrum of phenoxy-imine complex V-L^2	**Figure S8**
^{1}H NMR spectrum of phenoxy-amine complex Ti-L^3	**Figure S9**
^{13}C NMR spectrum of phenoxy-amine complex Ti-L^3	**Figure S10**
$M_{n\ NMR}$ and $M_{n\ theor}$ for PLA synthesized with vanadium complex V-L^2	**Table S3**
GPC curves of PLA synthesized with complexes Ti-L^1 and Ti-L^2	**Figure S11**
^{13}C NMR spectra of the PLA obtained in the presence of Ti-L^2 (a) and Ti-L^1 (b)	**Figure S12**
DSC thermograms of PLA synthesized with complexes Ti-L^2 and Ti-L^1	**Figure S13**
Results of ring opening polymerization of ε-caprolactone catalyzed by Ti-$L^{2,3}$ and V-L^2	**Table S4**
GPC curves of PCL synthesized with complex Ti-L^3	**Figure S14**
DSC thermograms of PCL synthesized with complex V-L^2	**Figure S15**
DSC thermograms of PCL synthesized with complex Ti-L^2 and Ti-L^3	**Figure S16**
Selected results of lactide polymerization with titanium and vanadium complexes	**Table S5**
Literature data on the polymerization of lactide in the presence of titanium complexes containing ligands with O and N donor atoms	**Table S6**

Crystallographic data

Table S1. Summary of crystal and refinement data for L^2H_2 and L^3H_2 proligands

	L^2H_2	L^3H_2
**Crystal data**		
Chemical formula	$C_{14}H_{13}NO_2$	$C_{22}H_{31}NO_2$
M_r	227.25	341.48
Crystal system, space group	Monoclinic, $P2_1/n$	Monoclinic, $P2_1/c$
Temperature (K)	293	100
a, *b*, *c* (Å)	4.5874 (4), 19.7695 (19), 12.6306 (10)	15.2211 (3), 10.76498 (12), 25.6076 (6)
β (°)	94.948 (7)	107.243 (2)
V (Å^3)	1141.21 (17)	4007.35 (14)
Radiation type	Mo *K*α	Cu *K*α
μ (mm^{-1})	0.09	0.56
Crystal size (mm)	0.3 × 0.25 × 0.2	0.4 × 0.3 × 0.2
**Data collection**		
Diffractometer	KM4 with Eos CCD	XtaLAB Synergy R, DW system, HyPix-Arc 150
T_{min}, T_{max}	0.797, 1.000	0.637, 1.000
No. of measured, independent and observed [$I > 2\sigma(I)$] reflections	3961, 2242, 1232	56704, 7977, 6542
R_{int}	0.032	0.033
$(\sin \theta/\lambda)_{max}$ (Å^{-1})	0.617	0.623
**Refinement**		
$R[F^2 > 2\sigma(F^2)]$, $wR(F^2)$, S	0.105, 0.320, 1.15	0.063, 0.181, 1.03
No. of reflections	2242	7977
No. of parameters	156	548
$\Delta\rho_{max}$, $\Delta\rho_{min}$ (e Å^{-3})	0.29, −0.27	0.60, −0.33

Table S2.Selected geometric parameters for compounds L^2H_2 and L^3H_2 (Å, º)

**L^2H_2**			
O1—C14	1.419 (7)	C2—C3	1.385 (8)
O1—H1	0.8200	C14—H14A	0.9700
O2—C2	1.348 (7)	C14—H14B	0.9700
O2—H2	0.8200	C12—C11	1.368 (9)
N1—C7	1.275 (7)	C12—H12	0.9300
N1—C8	1.422 (7)	C3—C4	1.377 (10)
C8—C13	1.391 (8)	C3—H3	0.9300
C8—C9	1.406 (8)	C11—C10	1.376 (9)
C9—C10	1.404 (8)	C11—H11	0.9300
C9—C14	1.499 (8)	C10—H10	0.9300
C1—C2	1.384 (8)	C4—C5	1.370 (10)
C1—C6	1.408 (8)	C4—H4	0.9300
C1—C7	1.440 (8)	C6—C5	1.374 (9)
C7—H7	0.9300	C6—H6	0.9300
C13—C12	1.367 (9)	C5—H5	0.9300
C13—H13	0.9300		
C14—O1—H1	109.5	O1—C14—H14B	109.0
C2—O2—H2	109.5	C9—C14—H14B	109.0
C7—N1—C8	122.2 (5)	H14A—C14—H14B	107.8
C13—C8—C9	119.4 (5)	C13—C12—C11	121.0 (6)
C13—C8—N1	124.9 (5)	C13—C12—H12	119.5
C9—C8—N1	115.7 (5)	C11—C12—H12	119.5
C10—C9—C8	118.7 (5)	C4—C3—C2	120.1 (6)
C10—C9—C14	121.1 (5)	C4—C3—H3	119.9
C8—C9—C14	120.2 (5)	C2—C3—H3	119.9
C2—C1—C6	118.7 (6)	C12—C11—C10	120.2 (6)
C2—C1—C7	122.5 (5)	C12—C11—H11	119.9
C6—C1—C7	118.7 (6)	C10—C11—H11	119.9
N1—C7—C1	122.0 (5)	C11—C10—C9	120.3 (6)
N1—C7—H7	119.0	C11—C10—H10	119.8
C1—C7—H7	119.0	C9—C10—H10	119.8
C12—C13—C8	120.3 (6)	C5—C4—C3	121.0 (7)
C12—C13—H13	119.8	C5—C4—H4	119.5
C8—C13—H13	119.8	C3—C4—H4	119.5
O2—C2—C1	121.8 (5)	C5—C6—C1	120.9 (7)
O2—C2—C3	118.3 (6)	C5—C6—H6	119.6
C1—C2—C3	119.9 (6)	C1—C6—H6	119.6
O1—C14—C9	113.1 (5)	C4—C5—C6	119.3 (7)
O1—C14—H14A	109.0	C4—C5—H5	120.3
C9—C14—H14A	109.0	C6—C5—H5	120.3
C7—N1—C8—C13	−2.1 (8)	C10—C9—C14—O1	19.8 (8)
C7—N1—C8—C9	175.8 (5)	C8—C9—C14—O1	−161.9 (5)
C13—C8—C9—C10	−0.6 (8)	C8—C13—C12—C11	1.0 (10)
N1—C8—C9—C10	−178.6 (5)	O2—C2—C3—C4	179.6 (6)

C13—C8—C9—C14	−179.0 (5)	C1—C2—C3—C4	0.3 (10)
N1—C8—C9—C14	3.0 (8)	C13—C12—C11—C10	−2.0 (10)
C8—N1—C7—C1	−178.1 (5)	C12—C11—C10—C9	1.7 (10)
C2—C1—C7—N1	−1.1 (9)	C8—C9—C10—C11	−0.3 (9)
C6—C1—C7—N1	178.0 (6)	C14—C9—C10—C11	178.0 (6)
C9—C8—C13—C12	0.3 (9)	C2—C3—C4—C5	−1.4 (11)
N1—C8—C13—C12	178.1 (6)	C2—C1—C6—C5	0.8 (10)
C6—C1—C2—O2	−179.3 (6)	C7—C1—C6—C5	−178.3 (6)
C7—C1—C2—O2	−0.2 (9)	C3—C4—C5—C6	2.2 (12)
C6—C1—C2—C3	−0.1 (9)	C1—C6—C5—C4	−1.9 (11)
C7—C1—C2—C3	179.0 (6)		
**L^3H_2**			
O1A—C2A	1.369 (2)	O2B—H2BA	0.8400
O1A—H1A	0.8400	C19B—C18B	1.374 (3)
O2A—C22A	1.443 (2)	C19B—C20B	1.386 (3)
O2A—H2A	0.8400	C19B—H19B	0.9500
N1A—C16A	1.405 (2)	C17B—C18B	1.375 (3)
N1A—C15A	1.463 (2)	C17B—H17B	0.9500
N1A—H1AA	0.8800	C4A—H4A	0.9500
C5B—C4B	1.399 (3)	C18B—H18B	0.9500
C5B—C6B	1.406 (3)	C22A—H22C	0.9900
C5B—C11B	1.529 (3)	C22A—H22D	0.9900
C1B—C2B	1.393 (3)	C8A—H8AA	0.9800
C1B—C6B	1.397 (3)	C8A—H8AB	0.9800
C1B—C15B	1.498 (3)	C8A—H8AC	0.9800
C11B—C13C	1.513 (4)	C9A—H9AA	0.9800
C11B—C14C	1.513 (4)	C9A—H9AB	0.9800
C11B—C12C	1.538 (3)	C9A—H9AC	0.9800
C11B—C12B	1.554 (7)	C10A—H10G	0.9800
C11B—C14B	1.573 (7)	C10A—H10H	0.9800
C11B—C13B	1.603 (6)	C10A—H10I	0.9800
C21A—C20A	1.378 (3)	C22B—O2C	1.518 (5)
C21A—C16A	1.410 (3)	C22B—H22A	0.9900
C21A—C22A	1.500 (3)	C22B—H22B	0.9900
N1B—C16B	1.391 (3)	C14A—H14G	0.9800
N1B—C15B	1.449 (2)	C14A—H14H	0.9800
N1B—H1B	0.8800	C14A—H14I	0.9800
C3B—C4B	1.386 (3)	C12A—H12G	0.9800
C3B—C2B	1.396 (3)	C12A—H12H	0.9800
C3B—C7B	1.540 (3)	C12A—H12I	0.9800
C6B—O1C	1.345 (3)	C20B—H20B	0.9500
C6B—H6B	0.9500	C13A—H13G	0.9800
C4B—H4B	0.9500	C13A—H13H	0.9800
C16A—C17A	1.396 (3)	C13A—H13I	0.9800
C18A—C17A	1.384 (3)	C12C—H12A	0.9800
C18A—C19A	1.386 (3)	C12C—H12B	0.9800
C18A—H18A	0.9500	C12C—H12C	0.9800
C2B—O1B	1.291 (4)	O1C—H1C	0.8400

C2B—H2B	0.9500	C13C—H13A	0.9800
C7A—C10A	1.529 (3)	C13C—H13B	0.9800
C7A—C8A	1.533 (3)	C13C—H13C	0.9800
C7A—C9A	1.536 (3)	C14C—H14A	0.9800
C7A—C3A	1.543 (2)	C14C—H14B	0.9800
C16B—C17B	1.396 (3)	C14C—H14C	0.9800
C16B—C21B	1.413 (3)	C8C—H8CA	0.9800
C7B—C8C	1.503 (4)	C8C—H8CB	0.9800
C7B—C10B	1.515 (5)	C8C—H8CC	0.9800
C7B—C9C	1.533 (5)	C9C—H9CA	0.9800
C7B—C10C	1.533 (5)	C9C—H9CB	0.9800
C7B—C8B	1.574 (6)	C9C—H9CC	0.9800
C7B—C9B	1.577 (6)	C10C—H10A	0.9800
C1A—C6A	1.388 (3)	C10C—H10B	0.9800
C1A—C2A	1.405 (2)	C10C—H10C	0.9800
C1A—C15A	1.503 (3)	C10B—H10D	0.9800
C2A—C3A	1.399 (3)	C10B—H10E	0.9800
C17A—H17A	0.9500	C10B—H10F	0.9800
C11A—C14A	1.521 (3)	C9B—H9BA	0.9800
C11A—C13A	1.533 (3)	C9B—H9BB	0.9800
C11A—C12A	1.536 (3)	C9B—H9BC	0.9800
C11A—C5A	1.538 (3)	C8B—H8BA	0.9800
C21B—C20B	1.382 (3)	C8B—H8BB	0.9800
C21B—C22B	1.504 (3)	C8B—H8BC	0.9800
C15A—H15C	0.9900	C13B—H13D	0.9800
C15A—H15D	0.9900	C13B—H13E	0.9800
C6A—C5A	1.394 (3)	C13B—H13F	0.9800
C6A—H6A	0.9500	C12B—H12D	0.9800
C5A—C4A	1.396 (3)	C12B—H12E	0.9800
C20A—C19A	1.395 (3)	C12B—H12F	0.9800
C20A—H20A	0.9500	C14B—H14D	0.9800
C3A—C4A	1.394 (3)	C14B—H14E	0.9800
C15B—H15A	0.9900	C14B—H14F	0.9800
C15B—H15B	0.9900	O1B—H1BA	0.8400
C19A—H19A	0.9500	O2C—H2C	0.8400
O2B—C22B	1.356 (3)		
C2A—O1A—H1A	109.5	C17B—C18B—H18B	119.7
C22A—O2A—H2A	109.5	O2A—C22A—C21A	113.24 (15)
C16A—N1A—C15A	120.54 (15)	O2A—C22A—H22C	108.9
C16A—N1A—H1AA	119.7	C21A—C22A—H22C	108.9
C15A—N1A—H1AA	119.7	O2A—C22A—H22D	108.9
C4B—C5B—C6B	116.67 (19)	C21A—C22A—H22D	108.9
C4B—C5B—C11B	121.33 (18)	H22C—C22A—H22D	107.7
C6B—C5B—C11B	121.98 (17)	C7A—C8A—H8AA	109.5
C2B—C1B—C6B	118.91 (18)	C7A—C8A—H8AB	109.5
C2B—C1B—C15B	119.8 (2)	H8AA—C8A—H8AB	109.5
C6B—C1B—C15B	121.24 (18)	C7A—C8A—H8AC	109.5
C13C—C11B—C14C	109.3 (2)	H8AA—C8A—H8AC	109.5

C13C—C11B—C5B	111.26 (19)	H8AB—C8A—H8AC	109.5
C14C—C11B—C5B	107.32 (19)	C7A—C9A—H9AA	109.5
C13C—C11B—C12C	108.2 (2)	C7A—C9A—H9AB	109.5
C14C—C11B—C12C	110.8 (2)	H9AA—C9A—H9AB	109.5
C5B—C11B—C12C	110.02 (19)	C7A—C9A—H9AC	109.5
C5B—C11B—C12B	109.9 (3)	H9AA—C9A—H9AC	109.5
C5B—C11B—C14B	115.3 (3)	H9AB—C9A—H9AC	109.5
C12B—C11B—C14B	106.1 (4)	C7A—C10A—H10G	109.5
C5B—C11B—C13B	114.4 (3)	C7A—C10A—H10H	109.5
C12B—C11B—C13B	106.3 (4)	H10G—C10A—H10H	109.5
C14B—C11B—C13B	104.1 (4)	C7A—C10A—H10I	109.5
C20A—C21A—C16A	118.94 (19)	H10G—C10A—H10I	109.5
C20A—C21A—C22A	120.81 (18)	H10H—C10A—H10I	109.5
C16A—C21A—C22A	120.20 (18)	O2B—C22B—C21B	115.6 (2)
C16B—N1B—C15B	120.41 (17)	C21B—C22B—O2C	112.2 (3)
C16B—N1B—H1B	119.8	C21B—C22B—H22A	109.2
C15B—N1B—H1B	119.8	O2C—C22B—H22A	109.2
C4B—C3B—C2B	116.46 (17)	C21B—C22B—H22B	109.2
C4B—C3B—C7B	121.18 (18)	O2C—C22B—H22B	109.2
C2B—C3B—C7B	122.35 (18)	H22A—C22B—H22B	107.9
O1C—C6B—C1B	117.5 (2)	C11A—C14A—H14G	109.5
O1C—C6B—C5B	121.2 (2)	C11A—C14A—H14H	109.5
C1B—C6B—C5B	121.23 (17)	H14G—C14A—H14H	109.5
C1B—C6B—H6B	119.4	C11A—C14A—H14I	109.5
C5B—C6B—H6B	119.4	H14G—C14A—H14I	109.5
C3B—C4B—C5B	124.38 (18)	H14H—C14A—H14I	109.5
C3B—C4B—H4B	117.8	C11A—C12A—H12G	109.5
C5B—C4B—H4B	117.8	C11A—C12A—H12H	109.5
C17A—C16A—N1A	122.67 (18)	H12G—C12A—H12H	109.5
C17A—C16A—C21A	119.46 (19)	C11A—C12A—H12I	109.5
N1A—C16A—C21A	117.83 (17)	H12G—C12A—H12I	109.5
C17A—C18A—C19A	120.8 (2)	H12H—C12A—H12I	109.5
C17A—C18A—H18A	119.6	C21B—C20B—C19B	122.8 (2)
C19A—C18A—H18A	119.6	C21B—C20B—H20B	118.6
O1B—C2B—C1B	117.2 (2)	C19B—C20B—H20B	118.6
O1B—C2B—C3B	120.4 (2)	C11A—C13A—H13G	109.5
C1B—C2B—C3B	122.29 (19)	C11A—C13A—H13H	109.5
C1B—C2B—H2B	118.9	H13G—C13A—H13H	109.5
C3B—C2B—H2B	118.9	C11A—C13A—H13I	109.5
C10A—C7A—C8A	107.33 (18)	H13G—C13A—H13I	109.5
C10A—C7A—C9A	107.87 (19)	H13H—C13A—H13I	109.5
C8A—C7A—C9A	110.38 (18)	C11B—C12C—H12A	109.5
C10A—C7A—C3A	111.68 (16)	C11B—C12C—H12B	109.5
C8A—C7A—C3A	109.31 (17)	H12A—C12C—H12B	109.5
C9A—C7A—C3A	110.22 (16)	C11B—C12C—H12C	109.5
N1B—C16B—C17B	122.12 (18)	H12A—C12C—H12C	109.5
N1B—C16B—C21B	119.80 (18)	H12B—C12C—H12C	109.5
C17B—C16B—C21B	117.98 (19)	C6B—O1C—H1C	109.5
C8C—C7B—C9C	108.6 (3)	C11B—C13C—H13A	109.5

C8C—C7B—C10C	113.0 (3)	C11B—C13C—H13B	109.5
C9C—C7B—C10C	106.3 (3)	H13A—C13C—H13B	109.5
C8C—C7B—C3B	108.42 (19)	C11B—C13C—H13C	109.5
C10B—C7B—C3B	111.3 (2)	H13A—C13C—H13C	109.5
C9C—C7B—C3B	112.1 (2)	H13B—C13C—H13C	109.5
C10C—C7B—C3B	108.5 (2)	C11B—C14C—H14A	109.5
C10B—C7B—C8B	108.0 (3)	C11B—C14C—H14B	109.5
C3B—C7B—C8B	114.7 (3)	H14A—C14C—H14B	109.5
C10B—C7B—C9B	107.7 (3)	C11B—C14C—H14C	109.5
C3B—C7B—C9B	108.3 (2)	H14A—C14C—H14C	109.5
C8B—C7B—C9B	106.4 (4)	H14B—C14C—H14C	109.5
C6A—C1A—C2A	119.07 (17)	C7B—C8C—H8CA	109.5
C6A—C1A—C15A	121.03 (15)	C7B—C8C—H8CB	109.5
C2A—C1A—C15A	119.86 (16)	H8CA—C8C—H8CB	109.5
O1A—C2A—C3A	119.43 (15)	C7B—C8C—H8CC	109.5
O1A—C2A—C1A	119.51 (17)	H8CA—C8C—H8CC	109.5
C3A—C2A—C1A	121.06 (16)	H8CB—C8C—H8CC	109.5
C18A—C17A—C16A	120.3 (2)	C7B—C9C—H9CA	109.5
C18A—C17A—H17A	119.9	C7B—C9C—H9CB	109.5
C16A—C17A—H17A	119.9	H9CA—C9C—H9CB	109.5
C14A—C11A—C13A	110.31 (19)	C7B—C9C—H9CC	109.5
C14A—C11A—C12A	108.5 (2)	H9CA—C9C—H9CC	109.5
C13A—C11A—C12A	106.9 (2)	H9CB—C9C—H9CC	109.5
C14A—C11A—C5A	109.96 (19)	C7B—C10C—H10A	109.5
C13A—C11A—C5A	108.82 (17)	C7B—C10C—H10B	109.5
C12A—C11A—C5A	112.27 (17)	H10A—C10C—H10B	109.5
C20B—C21B—C16B	118.6 (2)	C7B—C10C—H10C	109.5
C20B—C21B—C22B	120.37 (19)	H10A—C10C—H10C	109.5
C16B—C21B—C22B	121.05 (19)	H10B—C10C—H10C	109.5
N1A—C15A—C1A	110.68 (15)	C7B—C10B—H10D	109.5
N1A—C15A—H15C	109.5	C7B—C10B—H10E	109.5
C1A—C15A—H15C	109.5	H10D—C10B—H10E	109.5
N1A—C15A—H15D	109.5	C7B—C10B—H10F	109.5
C1A—C15A—H15D	109.5	H10D—C10B—H10F	109.5
H15C—C15A—H15D	108.1	H10E—C10B—H10F	109.5
C1A—C6A—C5A	122.05 (16)	C7B—C9B—H9BA	109.5
C1A—C6A—H6A	119.0	C7B—C9B—H9BB	109.5
C5A—C6A—H6A	119.0	H9BA—C9B—H9BB	109.5
C6A—C5A—C4A	116.80 (17)	C7B—C9B—H9BC	109.5
C6A—C5A—C11A	119.48 (16)	H9BA—C9B—H9BC	109.5
C4A—C5A—C11A	123.72 (19)	H9BB—C9B—H9BC	109.5
C21A—C20A—C19A	121.8 (2)	C7B—C8B—H8BA	109.5
C21A—C20A—H20A	119.1	C7B—C8B—H8BB	109.5
C19A—C20A—H20A	119.1	H8BA—C8B—H8BB	109.5
C4A—C3A—C2A	117.19 (16)	C7B—C8B—H8BC	109.5
C4A—C3A—C7A	121.90 (17)	H8BA—C8B—H8BC	109.5
C2A—C3A—C7A	120.91 (17)	H8BB—C8B—H8BC	109.5
N1B—C15B—C1B	111.86 (16)	C11B—C13B—H13D	109.5
N1B—C15B—H15A	109.2	C11B—C13B—H13E	109.5

C1B—C15B—H15A	109.2	H13D—C13B—H13E	109.5
N1B—C15B—H15B	109.2	C11B—C13B—H13F	109.5
C1B—C15B—H15B	109.2	H13D—C13B—H13F	109.5
H15A—C15B—H15B	107.9	H13E—C13B—H13F	109.5
C18A—C19A—C20A	118.7 (2)	C11B—C12B—H12D	109.5
C18A—C19A—H19A	120.7	C11B—C12B—H12E	109.5
C20A—C19A—H19A	120.7	H12D—C12B—H12E	109.5
C22B—O2B—H2BA	109.5	C11B—C12B—H12F	109.5
C18B—C19B—C20B	118.2 (2)	H12D—C12B—H12F	109.5
C18B—C19B—H19B	120.9	H12E—C12B—H12F	109.5
C20B—C19B—H19B	120.9	C11B—C14B—H14D	109.5
C18B—C17B—C16B	121.77 (19)	C11B—C14B—H14E	109.5
C18B—C17B—H17B	119.1	H14D—C14B—H14E	109.5
C16B—C17B—H17B	119.1	C11B—C14B—H14F	109.5
C3A—C4A—C5A	123.79 (19)	H14D—C14B—H14F	109.5
C3A—C4A—H4A	118.1	H14E—C14B—H14F	109.5
C5A—C4A—H4A	118.1	C2B—O1B—H1BA	109.5
C19B—C18B—C17B	120.6 (2)	C22B—O2C—H2C	109.5
C19B—C18B—H18B	119.7		
C4B—C5B—C11B—C13C	1.5 (3)	C15A—C1A—C2A—C3A	−179.35 (16)
C6B—C5B—C11B—C13C	−179.78 (18)	C19A—C18A—C17A—C16A	0.2 (3)
C4B—C5B—C11B—C14C	−118.0 (2)	N1A—C16A—C17A—C18A	−177.16 (18)
C6B—C5B—C11B—C14C	60.7 (2)	C21A—C16A—C17A—C18A	0.3 (3)
C4B—C5B—C11B—C12C	121.4 (2)	N1B—C16B—C21B—C20B	178.7 (2)
C6B—C5B—C11B—C12C	−59.9 (3)	C17B—C16B—C21B—C20B	2.2 (3)
C4B—C5B—C11B—C12B	66.1 (3)	N1B—C16B—C21B—C22B	−1.4 (3)
C6B—C5B—C11B—C12B	−115.2 (3)	C17B—C16B—C21B—C22B	−177.8 (2)
C4B—C5B—C11B—C14B	−174.0 (3)	C16A—N1A—C15A—C1A	172.41 (14)
C6B—C5B—C11B—C14B	4.7 (4)	C6A—C1A—C15A—N1A	132.04 (17)
C4B—C5B—C11B—C13B	−53.3 (4)	C2A—C1A—C15A—N1A	−50.1 (2)
C6B—C5B—C11B—C13B	125.4 (3)	C2A—C1A—C6A—C5A	−0.1 (3)
C2B—C1B—C6B—O1C	175.7 (2)	C15A—C1A—C6A—C5A	177.75 (16)
C15B—C1B—C6B—O1C	−7.3 (3)	C1A—C6A—C5A—C4A	1.7 (3)
C2B—C1B—C6B—C5B	−2.4 (3)	C1A—C6A—C5A—C11A	−178.32 (17)
C15B—C1B—C6B—C5B	174.52 (16)	C14A—C11A—C5A—C6A	−62.0 (3)
C4B—C5B—C6B—O1C	−175.7 (2)	C13A—C11A—C5A—C6A	58.9 (3)
C11B—C5B—C6B—O1C	5.5 (3)	C12A—C11A—C5A—C6A	177.0 (2)
C4B—C5B—C6B—C1B	2.4 (3)	C14A—C11A—C5A—C4A	117.9 (2)
C11B—C5B—C6B—C1B	−176.37 (16)	C13A—C11A—C5A—C4A	−121.1 (2)
C2B—C3B—C4B—C5B	−0.4 (3)	C12A—C11A—C5A—C4A	−3.0 (3)
C7B—C3B—C4B—C5B	178.86 (17)	C16A—C21A—C20A—C19A	−0.1 (3)
C6B—C5B—C4B—C3B	−1.0 (3)	C22A—C21A—C20A—C19A	177.36 (19)
C11B—C5B—C4B—C3B	177.79 (17)	O1A—C2A—C3A—C4A	−178.04 (16)
C15A—N1A—C16A—C17A	2.3 (3)	C1A—C2A—C3A—C4A	1.3 (3)
C15A—N1A—C16A—C21A	−175.17 (16)	O1A—C2A—C3A—C7A	2.6 (3)
C20A—C21A—C16A—C17A	−0.4 (3)	C1A—C2A—C3A—C7A	−178.08 (17)
C22A—C21A—C16A—C17A	−177.86 (17)	C10A—C7A—C3A—C4A	−1.1 (3)
C20A—C21A—C16A—N1A	177.22 (16)	C8A—C7A—C3A—C4A	−119.7 (2)

C22A—C21A—C16A—N1A	−0.3 (2)	C9A—C7A—C3A—C4A	118.8 (2)
C6B—C1B—C2B—O1B	−176.2 (2)	C10A—C7A—C3A—C2A	178.27 (19)
C15B—C1B—C2B—O1B	6.8 (3)	C8A—C7A—C3A—C2A	59.7 (2)
C6B—C1B—C2B—C3B	1.0 (3)	C9A—C7A—C3A—C2A	−61.8 (2)
C15B—C1B—C2B—C3B	−176.03 (16)	C16B—N1B—C15B—C1B	173.67 (19)
C4B—C3B—C2B—O1B	177.5 (2)	C2B—C1B—C15B—N1B	−67.9 (2)
C7B—C3B—C2B—O1B	−1.7 (3)	C6B—C1B—C15B—N1B	115.1 (2)
C4B—C3B—C2B—C1B	0.4 (3)	C17A—C18A—C19A—C20A	−0.7 (3)
C7B—C3B—C2B—C1B	−178.81 (17)	C21A—C20A—C19A—C18A	0.7 (3)
C15B—N1B—C16B—C17B	0.1 (3)	N1B—C16B—C17B—C18B	−179.5 (2)
C15B—N1B—C16B—C21B	−176.23 (19)	C21B—C16B—C17B—C18B	−3.2 (3)
C4B—C3B—C7B—C8C	−114.1 (3)	C2A—C3A—C4A—C5A	0.4 (3)
C2B—C3B—C7B—C8C	65.0 (3)	C7A—C3A—C4A—C5A	179.80 (18)
C4B—C3B—C7B—C10B	59.2 (3)	C6A—C5A—C4A—C3A	−1.9 (3)
C2B—C3B—C7B—C10B	−121.6 (3)	C11A—C5A—C4A—C3A	178.14 (18)
C4B—C3B—C7B—C9C	5.7 (3)	C20B—C19B—C18B—C17B	1.1 (4)
C2B—C3B—C7B—C9C	−175.1 (2)	C16B—C17B—C18B—C19B	1.5 (4)
C4B—C3B—C7B—C10C	122.8 (3)	C20A—C21A—C22A—O2A	121.2 (2)
C2B—C3B—C7B—C10C	−58.0 (3)	C16A—C21A—C22A—O2A	−61.4 (2)
C4B—C3B—C7B—C8B	−177.8 (3)	C20B—C21B—C22B—O2B	88.5 (3)
C2B—C3B—C7B—C8B	1.4 (4)	C16B—C21B—C22B—O2B	−91.4 (3)
C4B—C3B—C7B—C9B	−59.1 (3)	C20B—C21B—C22B—O2C	19.5 (4)
C2B—C3B—C7B—C9B	120.1 (3)	C16B—C21B—C22B—O2C	−160.5 (3)
C6A—C1A—C2A—O1A	177.88 (16)	C16B—C21B—C20B—C19B	0.3 (3)
C15A—C1A—C2A—O1A	0.0 (3)	C22B—C21B—C20B—C19B	−179.6 (2)
C6A—C1A—C2A—C3A	−1.5 (3)	C18B—C19B—C20B—C21B	−2.1 (4)

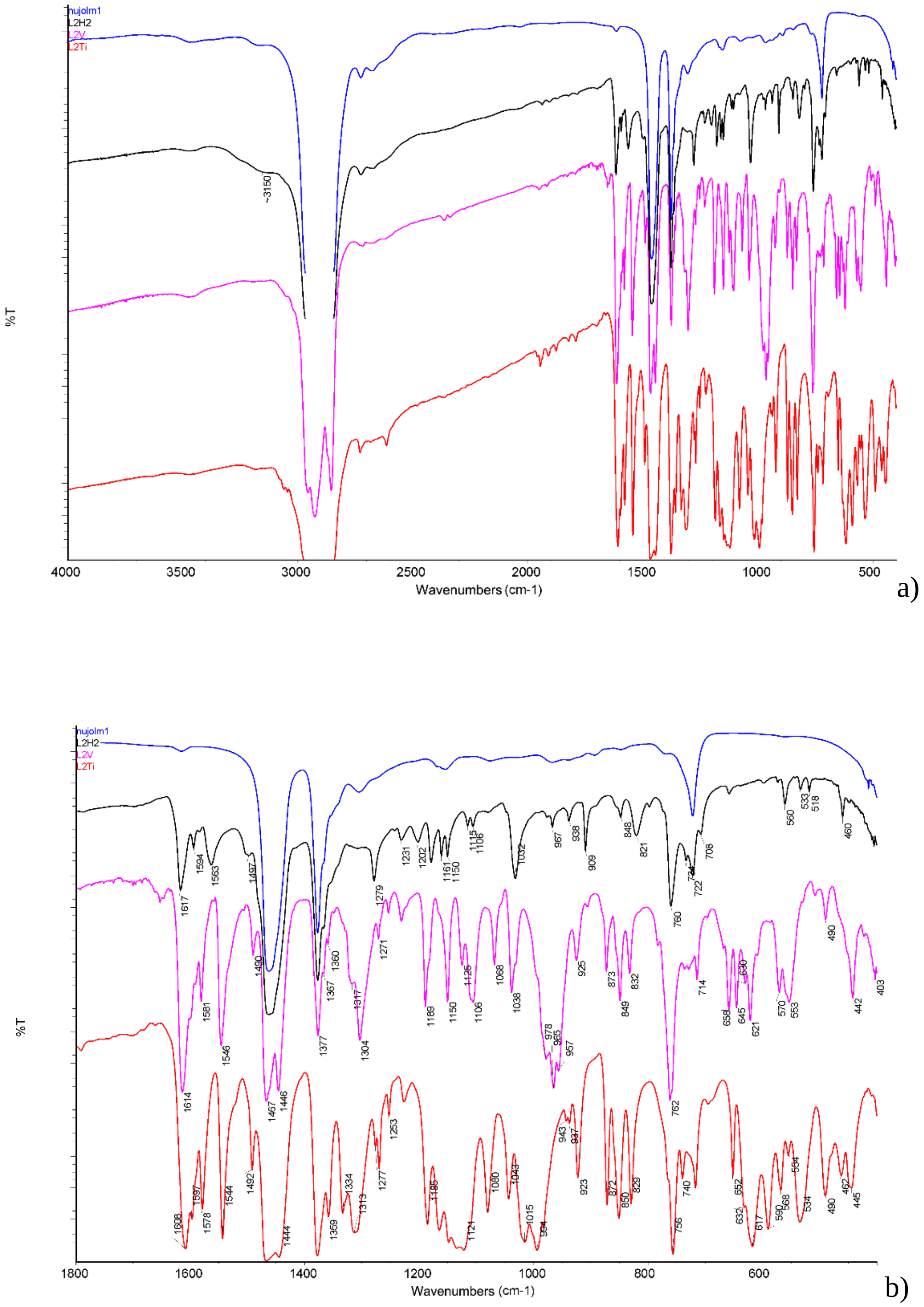

Figure S1. FTIR spectra of phenoxy-imine proligand L^2H_2 and corresponding titanium Ti-L^2 and vanadium V-L^2 complexes in nujol: full spectrum (a) and expanded 1800-400 cm^{-1} region (b).

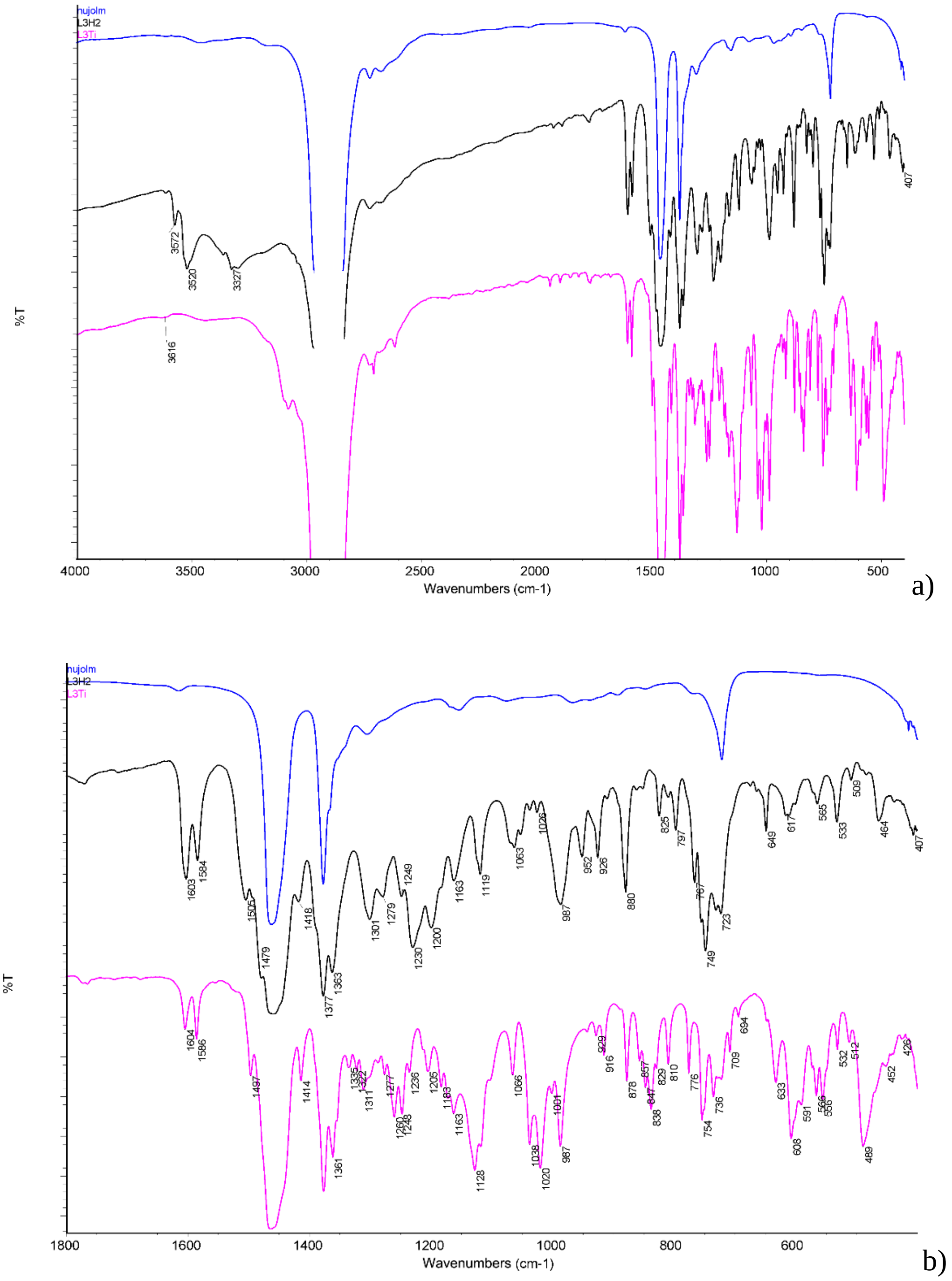

Figure S2. FTIR spectra of phenoxy-amine proligand L^3H_2 and corresponding titanium Ti-L^3 complex in nujol: full spectrum (a) and expanded 1800-400 cm^{-1} region (b).

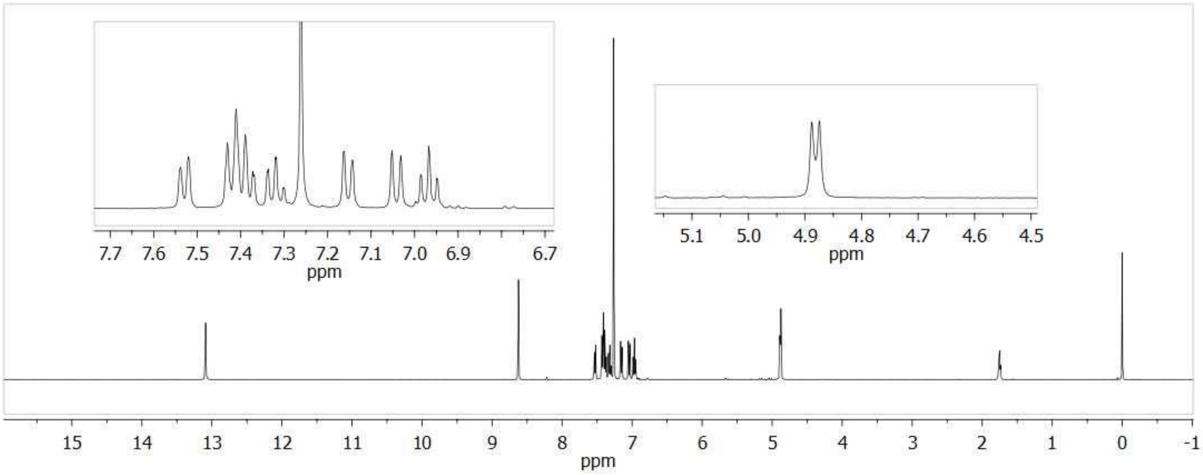

Figure S3. ^{1}H NMR spectrum of phenoxy-imine proligand $L^{2}H_{2}$ (chloroform-d1, room temperature).

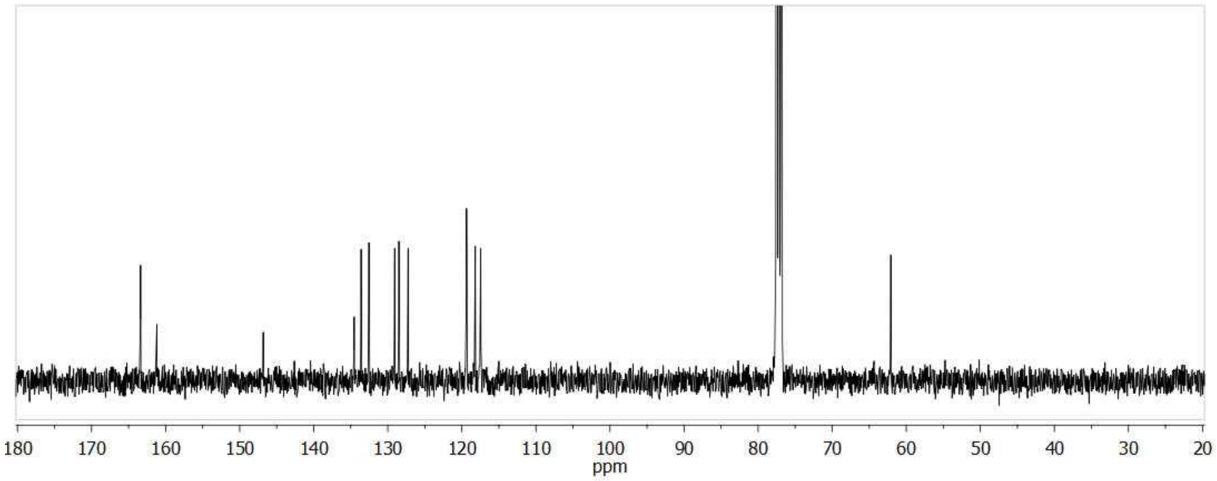

Figure S4. ^{13}C NMR spectrum of phenoxy-imine proligand $L^{2}H_{2}$ (chloroform-d1, room temperature).

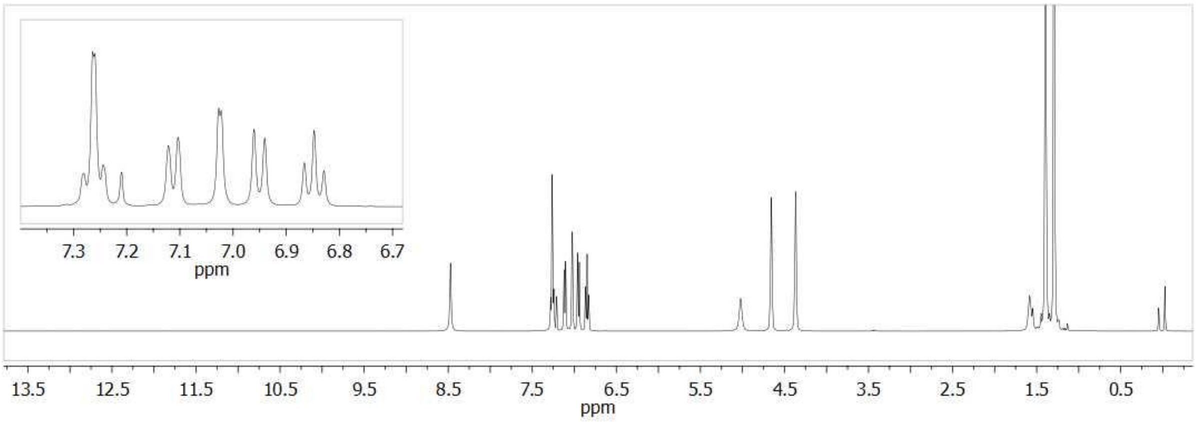

Figure S5. ^{1}H NMR spectrum of phenoxy-amine proligand $L^{3}H_{2}$ (chloroform-d1, room temperature).

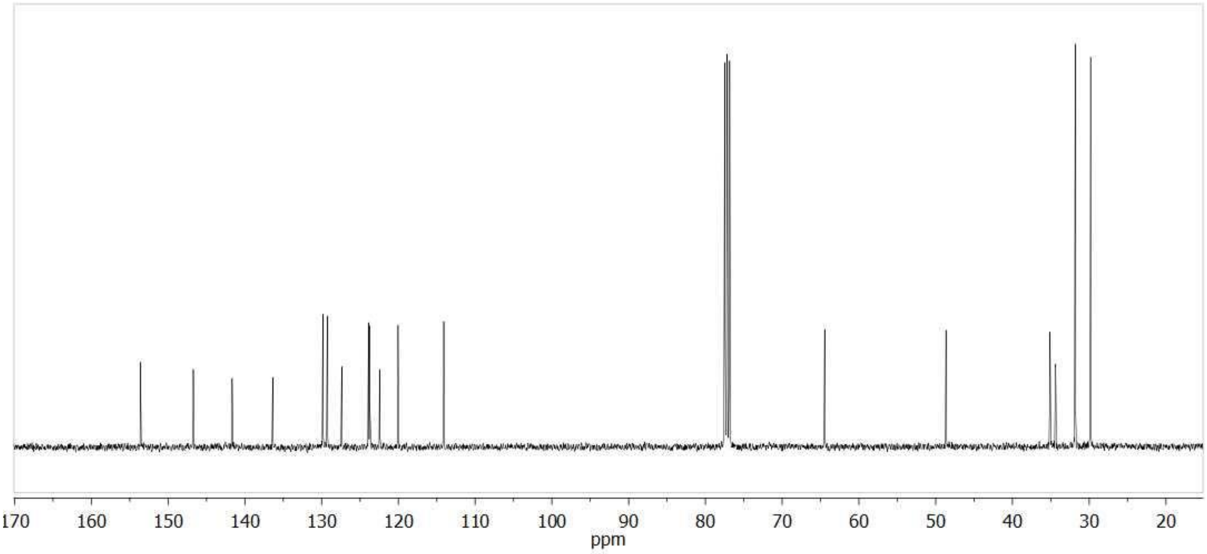

Figure S6. ^{13}C NMR spectrum of phenoxy-amine proligand L^3H_2 (chloroform-d1, room temperature).

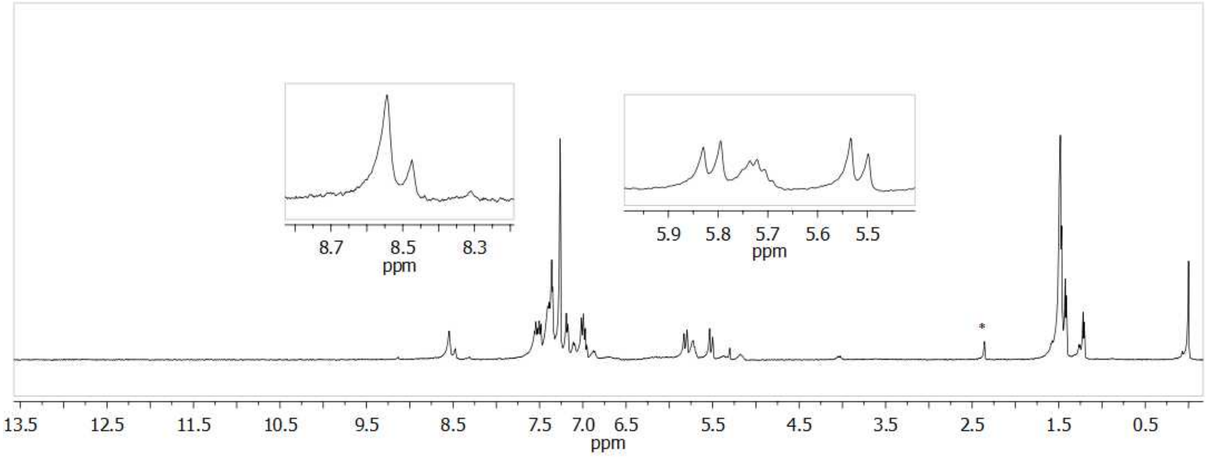

Figure S7. ^{1}H NMR spectrum of phenoxy-imine complex V-L^2 (chloroform-d1, room temperature),*toluene.

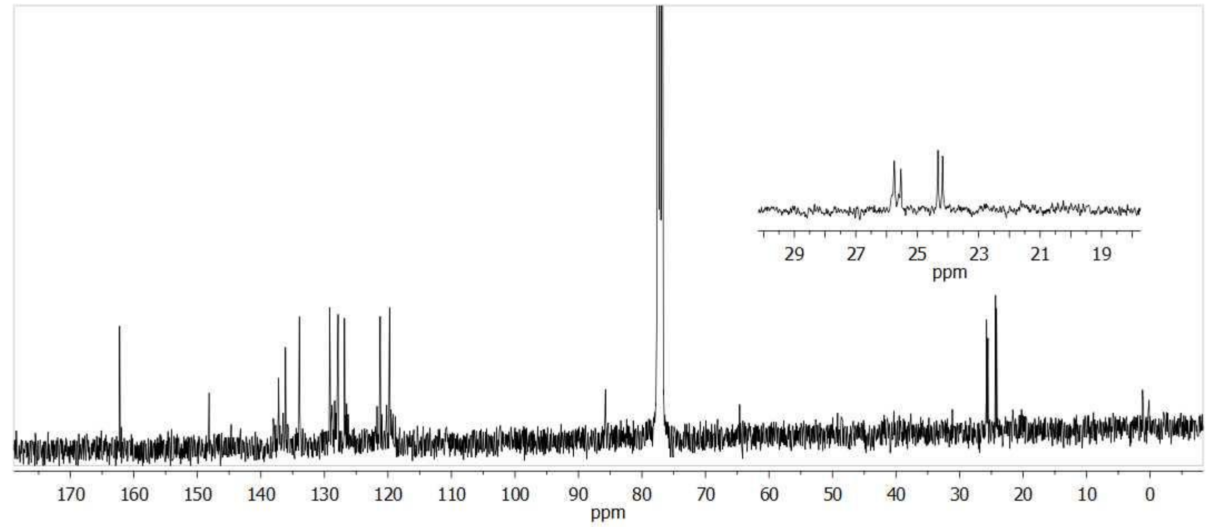

Figure S8. ^{13}C NMR spectrum of phenoxy-imine complex V-L^2 (chloroform-d1, room temperature).

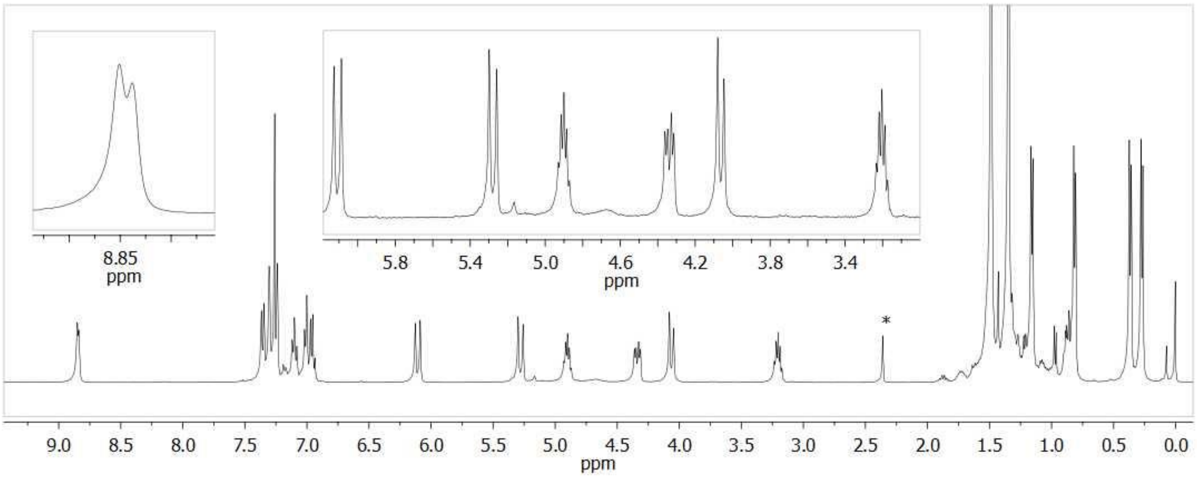

Figure S9. ^{1}H NMR spectrum of phenoxy-amine complex Ti-L^{3} (chloroform-d1, room temperature). *toluene

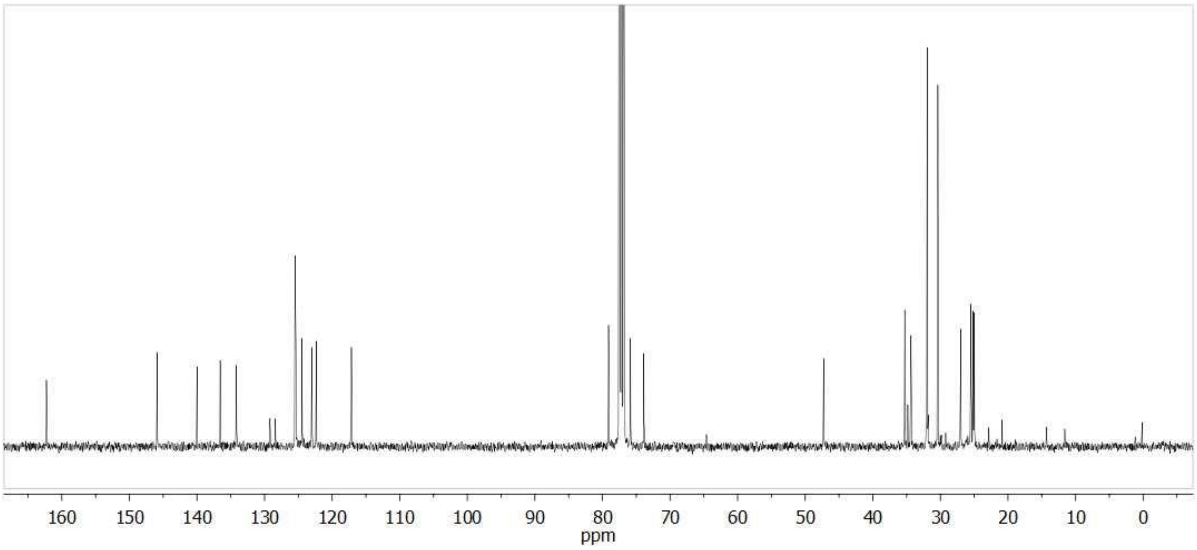

Figure S10. ^{13}C NMR spectrum of phenoxy-amine complex Ti-L^{3} (chloroform-d1, room temperature).

Table S3. $M_{n\ NMR}$ and $M_{n\ theo}$ for PLA synthesized with vanadium complex V-L[2]

Reaction time, min	$M_{n\ NMR}$, g/mol	$M_{n\ theo}$, g/mol
70	530	500
105	580	690
140	795	770
240	2100	1790

Conditions: [*rac*-LA]/[V] = 75, 145°C.

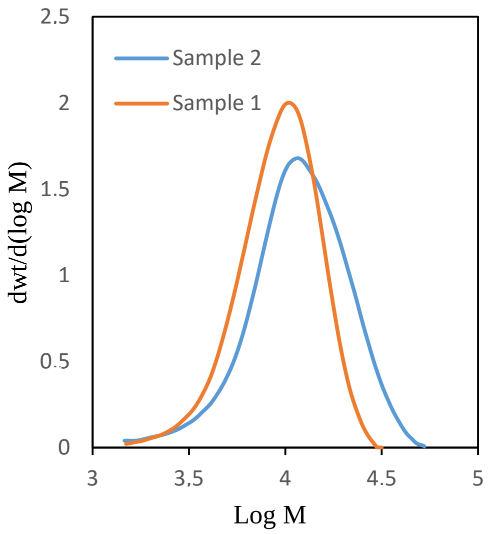

Sample	M_n	M_n×0.58[a)]	M_w/M_n
Sample 1	8100	4700	1.26
Sample 2	9800	5680	1.39

[a)]GPC values corrected with the coefficient 0.58 according to reference [47] in main text.

Figure S11. GPC curves of PLA synthesized with complexes Ti-L[1] (sample 1) and Ti-L[2] (sample 2). Reaction conditions: 145°C, [*rac*-LA]/[Ti] = 175.

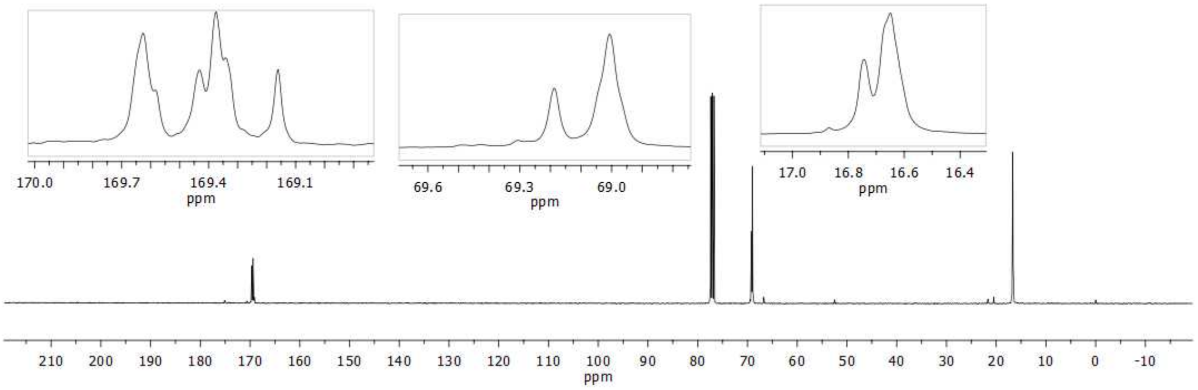

a)

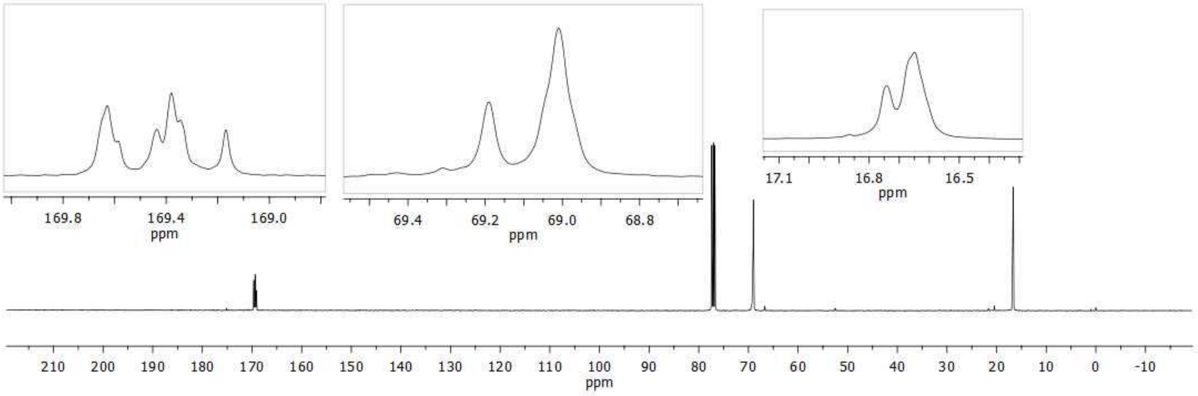

b)

Figure S12. ^{13}C NMR spectra of the PLA obtained in the presence of Ti-L^2 ([*rac*-LA]/[Ti] = 175, 145°C, 90 min) (a) and Ti-L^1 ([*rac*-LA]/[Ti] = 175, 145°C, 120 min (b).

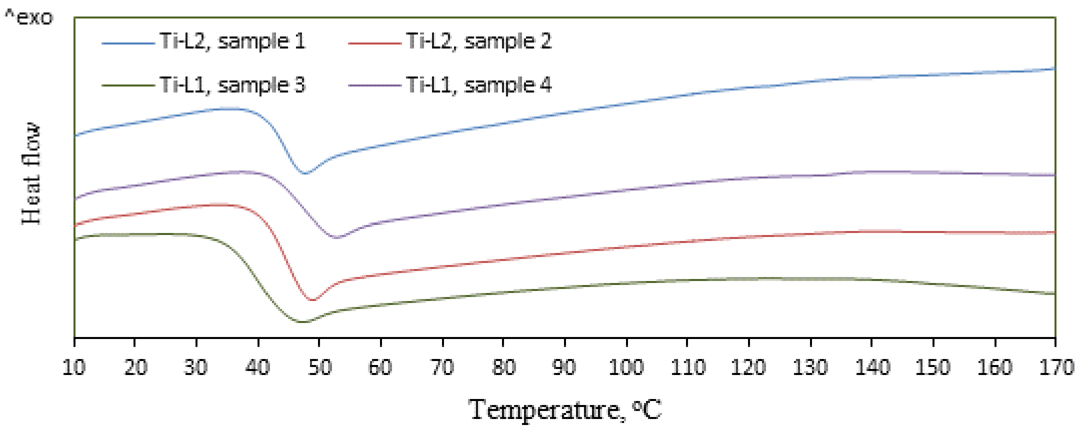

Figure S13. DSC thermograms of PLA synthesized with complex Ti-L^2 and Ti-L^1. Reaction conditions: 145°C, [*rac*-LA]/[Ti] = 175, 60 min (sample 1), 90 min (sample 2), 105 min (sample 3) and 75 min (sample 4).

Table S4. Results of ring opening polymerization of ε-caprolactone catalyzed by Ti-L^{2,3} and V- L^2

Complex	[ε-CL]/[M]	Reaction temp., °C	Reaction time, min	Monomer conversion, %	$M_{n\ NMR}$, g/mol	$M_{n\ theo}$, g/mol	$M_{n\ GPC}$, g/mol		$M_{n\ GPC}$ ×0.56[a)]	M_w/M_n
**Ti-L^2**	600	110	13	46.5	13500	15700	Peak 1	24600	13776	1.30
							Peak 2	750	420	1.22
		100	13	39.5	10800	13600	Peak 1	18700	10472	1.33
							Peak 2	720	403	1.29
		90	13	15.4	4600	5030	8650		4844	1.22
			25	35.1	10200	12100	-		-	-
**V-L^2**	600	110	130	9.0	5500	6140	-		-	-
			270	21.0	12500	16200	-		-	-
**Ti-L^3**	600	100	3	7.41	2730	2650	-		-	-
			4	18.9	5970	6590	-		-	-
			5	34.6	15110	11990	-		-	-
			6	52.9	19290	18210	-		-	-
			8	76.9	21330	26440	-		-	-

[a)]GPC values corrected with the coefficient 0.56 according to reference [31] in main text.

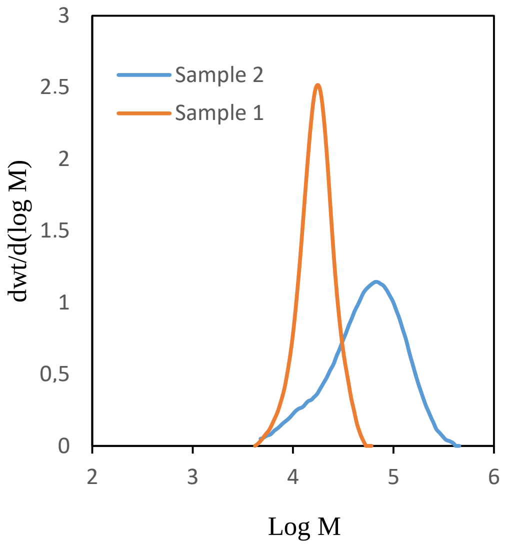

Sample	M_n	$M_n \times 0.56^{a)}$	M_w/M_n
Sample 1	15700	8792	1.17
Sample 2	38300	21448	1.99
[a] GPC values corrected with the coefficient 0.56 according to reference [31] in main text.			

Figure S14. GPC curves of PCL synthesized with complexes Ti-L[3]. Reaction conditions: [ε-CL]/[Ti] = 600, 100°C, 4 min (monomer conversion 18.9%, sample 1) and 6 min (monomer conversion 52.9%, sample 2).

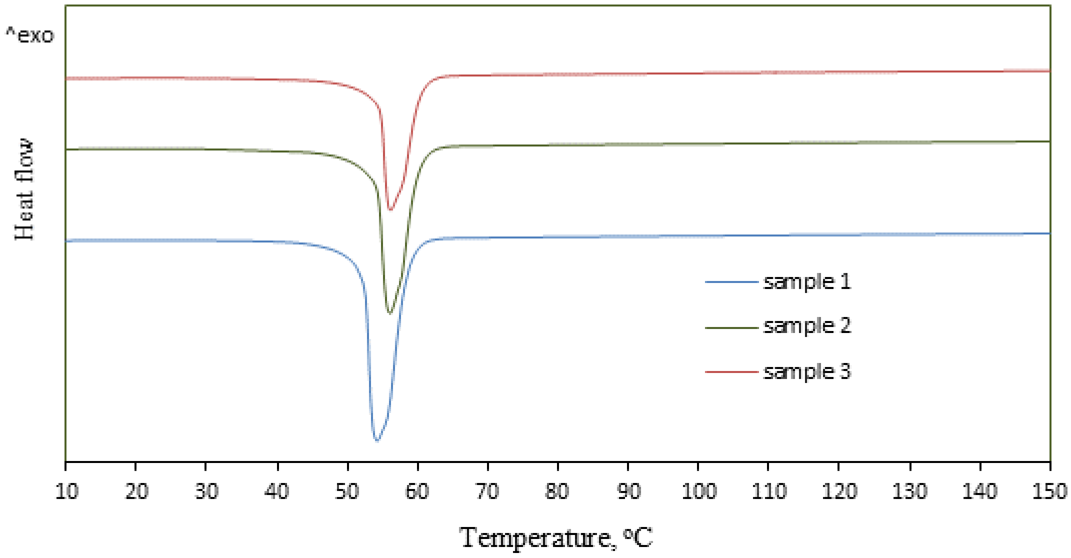

Figure S15. DSC thermograms of PCL synthesized with complex V-L[2]. Sample 1: [ε-CL]/[V] = 600, 110°C, 100 min. Sample 2: [ε-CL]/[V] = 600, 110°C, 360 min. Sample 3: [ε-CL]/[V] = 500, 120°C, 300 min.

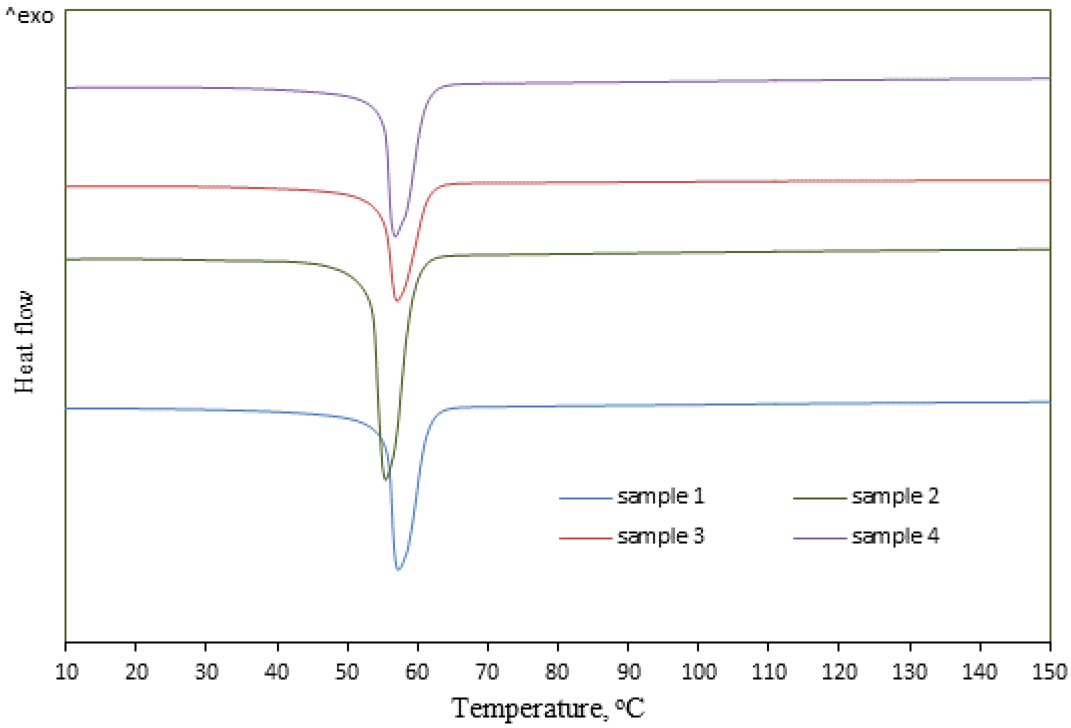

Figure S16. DSC thermograms of PCL synthesized with complexes Ti-L^2 and Ti-L^3. Sample 1: Ti-L^2, [ε-CL]/[Ti] = 600, 110°C, 13 min. Sample 2: Ti-L^2, [ε-CL]/[Ti] = 600, 90°C, 13 min. Sample 3: Ti-L^3, [ε-CL]/[Ti] = 600, 100°C, 8 min. Sample 4: Ti-L^3, [ε-CL]/[Ti] = 600, 100°C, 5 min.

Table S5. Selected results of lactide polymerization with titanium and vanadium complexes

Complex	Monomer	LA/Ti (V) molar ratio	Temp. (°C)	Time (min)	Conversion (%)	$M_{n\ NMR}$ (g/mol)
**Ti- L[1]**	*rac*-Lactide	175	145	45	26	3460
				60	40	4550
				75	46	6320
				90	58	6640
				105	61	8820
				120	66	8820
**Ti- L[2]**	*rac*-Lactide	175	145	30	45	4400
				50	56	7820
				60	69	-
				70	66.4	9360
				120	88	-
**Ti- L[3]**	*rac*-Lactide	175	145	8	3.4	164
				25	9.9	614
				60	42.7	3200
				80	61.1	4400
				100	68.8	4530
				120	78.0	5830
				130	81.4	5970
**Ti- L[3]**	*L*-Lactide	175	145	8	5.4	860
				16	9.1	1080
				25	10.8	1150
				40	18.0	1830
				60	29.9	3370
				80	44.2	4610
				100	57.5	5820
				120	69.0	7180
**V- L[2]**	*rac*-Lactide	175	145	90	3.30	-
				120	3.85	-
				150	4.76	-
				210	7.41	-
				240	8.26	-
				270	9.09	-

Table S6. Literature data on the polymerization of lactide in the presence of titanium complexes containing ligands with O and N donor atoms

Complex	Lactide monomer	LA/Ti molar ratio	Solvent	Temp. (°C)	Time	Conversion (%)	$M_{n\ GPC}$	M_w/M_n	Ref.
**Ti-1**	*L*-Lactide	200	-	130	3 h	99	16600	1.10	[1]
**Ti-1**	*L*-Lactide	400	-	130	3 h	84	21100	1.23	[1]
**Ti-2a**	*rac*-Lactide	200	-	140	35 min	95	25280	1.16	[2]
**Ti-2a**	*rac*-Lactide	400	-	100	75 min	92	52680	1.17	[2]
**Ti-2b**	*rac*-Lactide	200	-	140	25 min	91	27040	1.14	[2]
**Ti-3a**	*rac*-Lactide	100	toluene	80	750 min	96	4280	1.36	[3]
**Ti-3b**	*rac*-Lactide	100	toluene	80	1140 min	96	4440	1.26	[3]
**Ti-4**	*rac*-Lactide	100	toluene	80	24 h	82	20200	1.15	[4]
**Ti-5a**	*rac*-Lactide	200	-	140	29 min	100	52210	1.18	[5]
**Ti-5b**	*rac*-Lactide	200	-	140	32 min	100	51640	1.18	[5]
**Ti-6a**	*L*-Lactide	200	toluene	70	20 h	90	5900	1.30	[6]
**Ti-6b**	*L*-Lactide	200	toluene	70	20 h	74	5700	1.17	[6]
**Ti-7a**	*rac*-Lactide	100	-	130	8 h	87	10100	1.19	[7]
**Ti-7b**	*rac*-Lactide	100	-	130	5 h	94	5200	1.11	[7]
**Ti-8**	*L*-Lactide	100	-	140	15 min	11	-	-	[8]
**Ti-8**	*rac*-Lactide	100	-	140	15 min	95	13400	1.28	[8]
**Ti-9**	*rac*-Lactide	200	toluene	110	3 h	88	10500	1.19	[9]

References for Table S6:

1. Lai, F.J.; Huang, T.W.; Chang, Y.L.; Chang, H.Y.; Lu, W.Y.; Ding, S.; Chen, H.Y.; Chiu, C.C.; Wu, K.H. Titanium complexes bearing 2,6-Bis(o-hydroxyalkyl)pyridine ligands in the ring-opening polymerization of L-Lactide and ε-caprolactone. *Polymer* **2020**, *204*, 122860. https://doi.org/10.1016/j.polymer.2020.122860
2. *Roymuhury, S.K.;* Mandal, *M.;* Chakraborty, D.; Ramkumar, V. Homoleptic titanium and zirconium complexes exhibiting unusual O_{iminol}-metal coordination: Application in stereoselective ring-opening polymerization of lactide. Polym. Chem. **2021**, 12, 3953-3967. https://doi.org/10.1039/d1py00237f
3. Seo, C.C.Y.; Ahmed, M.; Oliver, A.G.; Durr, C.B. Titanium ONN-(phenolate) Alkoxide Complexes: Unique Reaction Kinetics for Ring-Opening Polymerization of Cyclic Esters. *Inorg. Chem.* **2021**, *60*, 19336-19344. https://doi.org/10.1021/acs.inorgchem.1c03157
4. Jeong, Y.; Shin, M.; Seo, M.; Kim, H. Ligand-Controlled Stereoselective Synthesis of Heterotactic Polylactide with Titanium(IV) Complexes. *Organometallics* **2022**, *41*, 328-334. https://doi.org/10.1021/acs.organomet.1c00666
5. Chakraborty, D.; Rajashekhar, B.; Mandal, M.; Ramkumar, V. Group 4 metal complexes containing the salalen ligands: Synthesis, structural characterization and studies on the ROP of cyclic esters. *J. Organometal. Chem.* **2018**, *871*, 111 – 121. https://doi.org/10.1016/j.jorganchem.2018.03.035
6. Ou, H.W.; Lu, W.Y.; Vandavasi, J.K.; Lin, Y.F.; Chen, H.Y.; Lin, C.C. Improvement in titanium complexes supported by Schiff bases in ring-opening polymerization of cyclic esters: ONO-tridentate Schiff bases. *Polymer* **2018**, *140*, 315-325. https://doi.org/10.1016/j.polymer.2018.02.016
7. Hu, M.; Zhang, W.; Ma, W.; Han, F.; Song W. Preparation of titanium complexes containing unsymmetric N2O2-ligands and their catalytic properties for polymerization of rac-lactide. *Polymer* **2018**, *153*, 445-452. https://doi.org/10.1016/j.polymer.2018.08.043
8. Wang, B.; Zhang, J.; Zhao, H.; Huang, H.; Zheng, J.; Wang, L.; Sun, J.; Zhang, Y.; Cao, Z. Titanium complexes bearing amine bis(phenolate) ligands: Synthesis, structure and catalysis in ring-opening polymerization of lactide. *Appl. Organometal. Chem.* **2017**, *31*, e3688. https://doi.org/10.1002/aoc.3688
9. Duan, R.; Qu, Z.; Pang, X.; Zhang, Y.; Sun, Z.; Zhang, H.; Bian, X.; Chen, X. Ring-Opening Polymerization of Lactide Catalyzed by Bimetallic Salen-Type Titanium Complexes. *Chin. J. Chem.* **2017**, *35*, 640-644. https://doi.org/10.1002/cjoc.201600580

Titanium complexes included in Table S6:

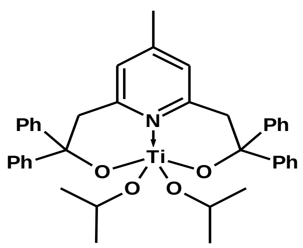

Ti-1 [1]

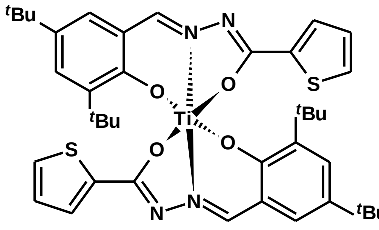

Ti-2a [2]

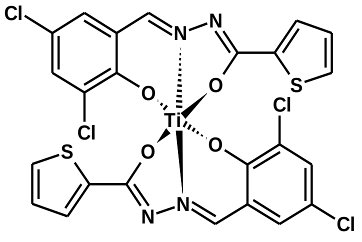

Ti-2b [2]

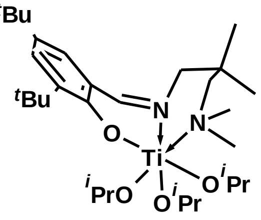

Ti-3a [3]

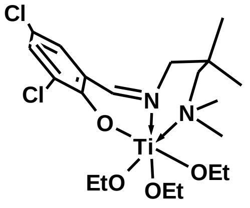

Ti-3b [3]

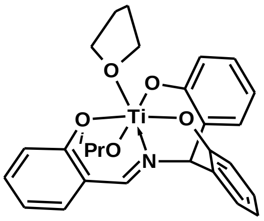

Ti-4 [4]

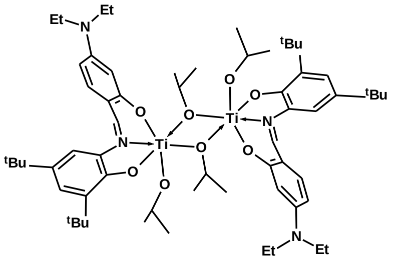

Ti-6a [6]

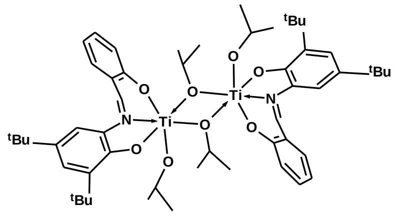

Ti-6b [6]

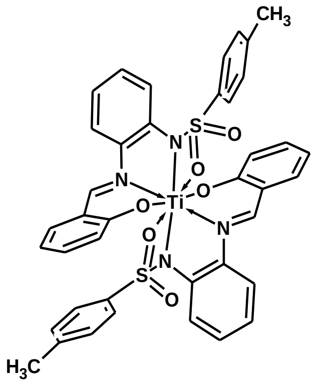

Ti-5a [5]

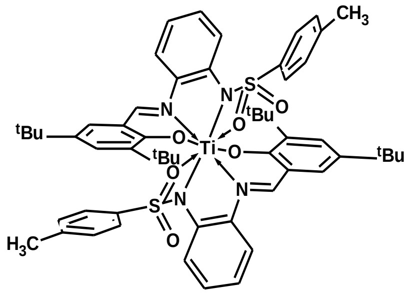

Ti-5b [5]

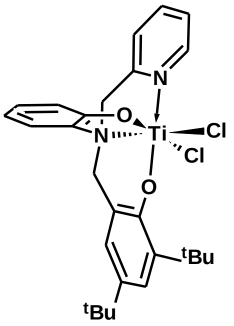

7a [7]

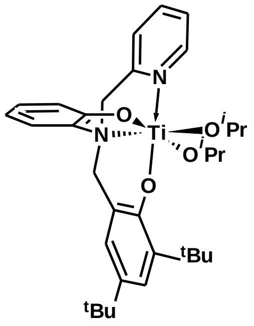

7b [7]

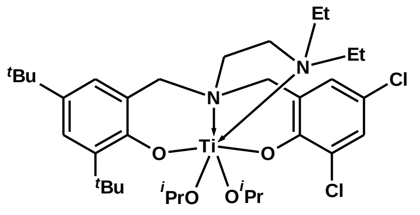

Ti-8 [8]

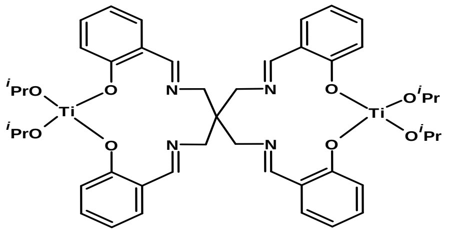

Ti-9 [9]
